# Brain inflammation co-localizes highly with tau in mild cognitive impairment due to early-onset Alzheimer’s disease

**DOI:** 10.1093/brain/awae234

**Published:** 2024-07-16

**Authors:** Johanna Appleton, Quentin Finn, Paolo Zanotti-Fregonara, Meixiang Yu, Alireza Faridar, Mohammad O Nakawah, Carlos Zarate, Maria C Carrillo, Bradford C Dickerson, Gil D Rabinovici, Liana G Apostolova, Joseph C Masdeu, Belen Pascual

**Affiliations:** Nantz National Alzheimer Center, Stanley H. Appel Department of Neurology, Houston Methodist Research Institute, Weill Cornell Medicine, Houston, TX 77030, USA; Nantz National Alzheimer Center, Stanley H. Appel Department of Neurology, Houston Methodist Research Institute, Weill Cornell Medicine, Houston, TX 77030, USA; Molecular Imaging Branch, National Institute of Mental Health, NIH, Bethesda, MD 20892, USA; Cyclotron and Radiopharmaceutical Core, Houston Methodist Research Institute, Weill Cornell Medicine, Houston, TX 77030, USA; Nantz National Alzheimer Center, Stanley H. Appel Department of Neurology, Houston Methodist Research Institute, Weill Cornell Medicine, Houston, TX 77030, USA; Nantz National Alzheimer Center, Stanley H. Appel Department of Neurology, Houston Methodist Research Institute, Weill Cornell Medicine, Houston, TX 77030, USA; Nantz National Alzheimer Center, Stanley H. Appel Department of Neurology, Houston Methodist Research Institute, Weill Cornell Medicine, Houston, TX 77030, USA; Medical & Scientific Relations Division, Alzheimer's Association, Chicago, IL 60603, USA; Department of Neurology, Massachusetts General Hospital, Boston, MA 02114, USA; Department of Neurology, University of California San Francisco, San Francisco, CA 94143, USA; Department of Neurology, Indiana University School of Medicine, Indianapolis, IN 46202, USA; Nantz National Alzheimer Center, Stanley H. Appel Department of Neurology, Houston Methodist Research Institute, Weill Cornell Medicine, Houston, TX 77030, USA; Nantz National Alzheimer Center, Stanley H. Appel Department of Neurology, Houston Methodist Research Institute, Weill Cornell Medicine, Houston, TX 77030, USA

**Keywords:** inflammation, mild cognitive impairment, early-onset Alzheimer’s disease, ^11^C-ER176 PET, TSPO

## Abstract

Brain inflammation, with an increased density of microglia and macrophages, is an important component of Alzheimer’s disease and a potential therapeutic target. However, it is incompletely characterized, particularly in patients whose disease begins before the age of 65 years and, thus, have few co-pathologies. Inflammation has been usefully imaged with translocator protein (TSPO) PET, but most inflammation PET tracers cannot image subjects with a low-binder *TSPO* rs6971 genotype. In an important development, participants with any *TSPO* genotype can be imaged with a novel tracer, ^11^C-ER176, that has a high binding potential and a more favourable metabolite profile than other TSPO tracers currently available. We applied ^11^C-ER176 to detect brain inflammation in mild cognitive impairment (MCI) caused by early-onset Alzheimer’s disease. Furthermore, we sought to correlate the brain localization of inflammation, volume loss, elevated amyloid-β (Aβ)and tau.

We studied brain inflammation in 25 patients with early-onset amnestic MCI (average age 59 ± 4.5 years, 10 female) and 23 healthy controls (average age 65 ± 6.0 years, 12 female), both groups with a similar proportion of all three TSPO-binding affinities. ^11^C-ER176 total distribution volume (*V*_T_), obtained with an arterial input function, was compared across patients and controls using voxel-wise and region-wise analyses. In addition to inflammation PET, most MCI patients had Aβ (*n* = 23) and tau PET (*n* = 21). For Aβ and tau tracers, standard uptake value ratios were calculated using cerebellar grey matter as region of reference. Regional correlations among the three tracers were determined. Data were corrected for partial volume effect. Cognitive performance was studied with standard neuropsychological tools.

In MCI caused by early-onset Alzheimer’s disease, there was inflammation in the default network, reaching statistical significance in precuneus and lateral temporal and parietal association cortex bilaterally, and in the right amygdala. Topographically, inflammation co-localized most strongly with tau (*r* = 0.63 ± 0.24). This correlation was higher than the co-localization of Aβ with tau (*r* = 0.55 ± 0.25) and of inflammation with Aβ (0.43 ± 0.22). Inflammation co-localized least with atrophy (−0.29 ± 0.26). These regional correlations could be detected in participants with any of the three rs6971 *TSPO* polymorphisms. Inflammation in Alzheimer’s disease-related regions correlated with impaired cognitive scores.

Our data highlight the importance of inflammation, a potential therapeutic target, in the Alzheimer’s disease process. Furthermore, they support the notion that, as shown in experimental tissue and animal models, the propagation of tau in humans is associated with brain inflammation.

## Teaser

Using a novel PET tracer for imaging inflammation, Appleton *et al.* show that people with mild cognitive impairment with onset before the age of 65 years show brain inflammation that co-localizes with tau and correlates with cognitive impairment.

## Introduction

Microglia and astrocytes play a major role in the Alzheimer’s disease (AD) process^1,2^; these cells interact with amyloid-β (Aβ) and phosphorylated tau and have multiple functions, including neurotoxicity and neuroprotection, at various stages of the disease and at various brain locations.^3,4^ Microglia and astrocytes in human AD have been usefully studied in post-mortem tissue^5^ with anatomic specificity but not allowing for longitudinal follow-up in the same individual; related biomarkers have been studied in CSF^6^ or plasma,^7^ yielding longitudinal data but no anatomic localization in the brain. Providing both anatomic localization and the possibility of longitudinal follow-up, PET has been used extensively to study microglia and astrocytes in AD.^8,9^

Early-onset AD (EOAD), presenting before the age of 65, has been observed to be a ‘purer’ form of the disease, with early-onset patients having a higher tau burden and fewer co-pathologies than late-onset patients.^10-13^ Therefore, investigating inflammation in a cohort of EOAD patients could help elucidate more clearly the relationship between brain inflammation and the build-up of Aβ and hyperphosphorylated tau. Although some inflammation PET studies have included patients with EOAD,^14-20^ none has centred so far on the study of inflammation in mild cognitive impairment (MCI) caused by EOAD and its correlation with Aβ and tau build-up in the brain.

The most common target of inflammation PET is an 18 kDa translocator protein, TSPO, that reflects the regional density of microglia, peripherally derived macrophages, astrocytes and endothelial cells in the human brain.^21,22^ TSPO imaging has provided highly anatomically specific information, not only in AD but also in other neurodegenerative diseases, for instance showing a signal in motor cortex in amyotrophic lateral sclerosis.^23,24^ The first generation TSPO tracer, ^11^C-PK11195, has a low binding affinity (BP_ND_), which limits its sensitivity.^25^ Second-generation tracers have improved binding affinities, evidencing that the rs6971 polymorphism of the *TSPO* gene causes differences in the binding affinity of the tracers, categorizing individuals as high-, mixed- or low-affinity binders. Most of the second generation TSPO PET tracers cannot be used to image low-affinity binders, which account for approximately 10% of the population.^26^ Furthermore, for ^11^C-PBR28, the most frequently used second generation tracer, the BP_ND_ differs markedly in high- as compared to mix-affinity binders (ratio 2.5),^27^ prompting some researchers to study only high-affinity binders,^28,29^ which make up only about 49% of predominantly Caucasian populations.^30^ This serious shortcoming is addressed by ^11^C-ER176, a novel PET tracer with a high affinity for TSPO, allowing for the robust imaging of even low-affinity binders.^25,31,32^ In a previous study, we found the BP_ND_ of ^11^C-ER176 to be more than four times larger than that of ^11^C-PBR28 for high-affinity binders and more than nine times larger for mixed-affinity binders.^33^ However, the effectiveness of this tracer to study microglia and astrocytes in AD has not been determined. We hypothesized that ^11^C-ER176 would allow for the detection of a TSPO signal in AD patients with any variant of the rs6971 *TSPO* polymorphism.

Nomenclature related to microglia is in a state of flux. An expert panel has suggested that authors define clearly how they use terms related to microglia.^34^ The term ‘neuroinflammation', commonly used in reports of human AD,^9^ is now discouraged.^34^ Since a more convenient term is not yet universally accepted and the TSPO signal does not reflect a single cell type, to denote the density of microglia, macrophages, astrocytes and endothelial cells reflected by the TSPO PET signal, we will use the term ‘inflammation.’ With this term, we do not intend to indicate that these cells are in a neurotoxic state, because the TSPO PET signal does not allow for the definition of the state of microglia.^9,21^ Additionally, when referencing ‘tau,’ it pertains to abnormal, hyperphosphorylated tau aggregates, not the native tau protein.

To clarify the relationship in the brain among inflammation, atrophy, Aβ and tau, we measured inflammation in a cohort of EOAD patients using ^11^C-ER176 and an arterial input function. As at advanced stages of the disease the widespread pathology may obscure on PET the topographic relationships among inflammation, Aβ and tau, we chose a cohort of patients with MCI caused by EOAD (MCI-EOAD).

## Materials and methods

### Participants

Participants (*n* = 48) were recruited at the Houston Methodist Nantz National Alzheimer Center and included 25 patients diagnosed with MCI-EOAD (average age 59 ± 4.5, 10 female) and, as controls, 23 cognitively unimpaired (CU) individuals (average age 65 ± 6.0, 12 female) with similar sex distribution but slightly older than the patients (Table 1). MCI-EOAD diagnosis was based on clinical presentation, MRI and either a positive Aβ (*n* = 23) and tau (*n* = 21) PET or a fluorodeoxyglucose PET (*n* = 1) or Aβ/tau ratio in CSF (*n* = 1) diagnostic of AD. All patients had a Clinical Dementia Rating (CDR)^35^ score of 0.5. Controls were considered cognitively unimpaired based on clinical and neuropsychological screening, normal MRI and absence of neurological, psychiatric or other major medical illnesses. To limit their radiation exposure, CU controls did not have Aβ or tau imaging. Therefore, for group comparisons we selected 19 CU participants (age 63 ± 7.5, 12 female) from the Alzheimer’s Disease Neuroimaging Initiative (ADNI) studied with the same Aβ PET tracer, ^18^F-florbetaben, and in the same scanner model as our patients, and 23 CU participants (age 62 ± 6.4, 15 female) from in-house Institutional Review Board-approved protocols who had undergone ^18^F-flortaucipir PET imaging.

**Table 1:** Demographics and neuropsychological test performance

	MCI-EOAD	CU	
	*n* = 25	*n* = 23	*P*-value
Female/male^a^	10/15	12/11	n.s.
Age, years (mean ± SD)	59.2 ± 4.5	64.9 ± 6.0	<0.01
Years of Education (mean ± SD)	16.5 ± 2.1	17.3 ± 4.3	n.s.
MMSE (mean ± SD)	22.0 ± 4.6	29.3 ± 1.2	<0.001
DemTect Total Score (mean ± SD)	8.4 ± 4.3	17.2 ± 1.4	<0.001
DemTect Immediate Recall (mean ± SD)	8.8 ± 3.2	14.0 ± 2.2	<0.001
DemTect Delayed Recall (mean ± SD)	1.7 ± 2.0	6.3 ± 2.1	<0.001
Clock Drawing Test (mean ± SD)	9.4 ± 2.2	12.6 ± 1.2	<0.001
ACE: Naming (mean ± SD)	10.8 ± 2.1	12.0 ± 0.2	<0.05
Aβ PET (florbetaben/florbetapir/PIB)	21/1/1	Not performed	–
^18^F-flortaucipir PET	21	Not performed	–
*TSPO* high-/mixed-/low-affinity binders^a,b^	12/8/5	9/10/4	n.s.

ACE = Addenbrooke’s Cognitive Examination; CU = cognitive unimpaired; MCI-EOAD = mild cognitive impairment caused by early-onset Alzheimer’s disease; MMSE = Mini-Mental Status Examination; n.s. = no statistically significant differences; PIB = Pittsburgh compound B; SD = standard deviation; TSPO = translocator protein.

^a^No statistically significant differences between patients and controls, as assessed by chi-squared test.

^b^Determined by *TSPO* Ala147Thr (rs6971) polymorphism genotyping.

For all 48 participants having inflammation imaging with ^11^C-ER176, the *TSPO* rs6971 polymorphism was determined using the TaqMan assay (Applied Biosystems). *TSPO* genotypes did not differ statistically between patients and controls (Table 1). Procedures were approved by the Methodist Hospital Research Institute Committee on Human Research, and written informed consent was obtained from all participants according to the Declaration of Helsinki.

### Neuropsychological evaluation

All 48 inflammation participants underwent an initial neuropsychological screening that included four measures (Table 1). The Mini-Mental State Examination (MMSE)^36^ and DemTect^37^ explored several cognitive domains, including verbal memory, executive function, attention and language. The Clock Drawing Test^38^ was used to evaluate visuospatial constructional abilities. The Addenbrooke’s Cognitive Examination^39^ provided a measure of semantic impairment, naming and comprehension. In addition to the initial screening, 21 of 25 patients were further evaluated with the neuropsychological battery of the National Alzheimer’s Coordinating Center (NACC) Uniform Data Set (UDS v3.0),^40^ through the Longitudinal Early-onset Alzheimer Disease Study (LEADS)^41^ (Supplementary Table 1).

### MRI acquisition and processing

The 48 inflammation participants were imaged with the same 3 T Siemens Vida MRI scanner (Siemens Medical Solutions), except for eight participants imaged on a 3 T Philips Ingenia MRI scanner (Philips Medical Systems) and three participants imaged on a 3 T Siemens Skrya scanner (Siemens Medical Solutions). The protocol included a 3D T1-weighted sequence for cortical thickness measurements and PET anatomical co-registration, tissue segmentation (grey and white matter along with CSF) and parcellation of the regions of interest. The parameters for the T1-weighted sequence acquired had slight differences across scanners in flip angle, as well as repetition time and echo time (Supplementary Table 2). These differences are expected to have little or no effect on the comparison of cortical thickness and none on the comparison of PET data across groups.

Cortical thickness was computed for the ^11^C-ER176 cohort with the Freesurfer Software Package version 7 pipeline (Athinoula A. Martinos Center for Biomedical Imaging, Boston). The technical details have been described in detail.^42,43^ A Gaussian kernel of 10 mm full-width at half-maximum (FWHM) was applied to the subjects’ cortical thickness maps before further analyses. Parameters for the MRIs acquired to facilitate PET processing for the ^18^F-florbetaben and ^18^F-flortaucipir CU control groups are described in Supplementary Table 2.

### PET image acquisition and processing

^11^C-ER176, ^18^F-florbetaben and ^18^F-flortaucipir PET scans were performed at the Houston Methodist Research Institute PET Core facility. Of the 25 MCI-EOAD patients, 21 underwent ^18^F-florbetaben and ^18^F-flortaucipir PET imaging through the LEADS study. One patient had a ^11^C-PIB PET scan and a second patient a clinical ^18^F-florbetapir PET scan. Supplementary Table 2 lists participant scanner allocation, scanner resolution and other specifications. PET imaging resolution was harmonized by applying a scanner-specific smoothing filter.^44^ Additional smoothing was applied to PET images from our higher resolution scanners to lower their FWHM to the nominal resolution of the GE Discovery scanner. Modelling the point spread function (PSF) of our scanners as a Gaussian filter, further smoothing gave a net FWHM by the following formula:


(1) $$FWHM_{net}^{2}=FWHM_{additional}^{2}+FWHM_{PSF}^{2}.$$

Setting *FWHM_net_* to the resolution of the GE Discovery scanner allowed us to solve for the necessary additional smoothing kernel. This method gives similar results when applied to the scanners measured experimentally for the ADNI project.^44^ As in previous studies using data from various scanners^45^ and considering the preprocessing harmonization procedure described above, scanner was not included as a covariate in the statistical analyses. The number of scanners in this study compares favourably with the number of scanners in ADNI^46^ or similar image databases.

Although ^11^C-ER176, ^18^F-florbetaben and ^18^F-flortaucipir PET imaging acquisition and processing followed different pipelines, several steps were common to the three tracers. Participants were comfortably positioned on the scanner bed, with head movement restricted by a thermoplastic mask. Before each PET scan, a low-dose CT scan was acquired for attenuation correction. After PET acquisition, motion correction and co-registration between PET and MRI was performed with PMOD v.3.9 (PMOD Technologies LLC, Zurich, Switzerland). ^11^C-ER176, ^18^F-florbetaben and ^18^F-flortaucipir PET images were processed at both voxel and regional levels.

At the regional level, we followed two different procedures. First, in order to determine the tau pathology spread of our MCI-EOAD patients and the pattern of inflammation in the same areas, we compared the spatial distribution of ^11^C-ER176 and ^18^F-flortaucipir through the Tau-PET Braak stages, as previously described using the Desikan–Killiany–Tourville atlas segmentation.^47^ We modified the method of Pascoal *et al*.,^48^ by combining Braak regions I and II, and so we defined the following regions of interest (ROIs): Braak I-II (transentorhinal, entorhinal and hippocampus); Braak III (amygdala, parahippocampal gyrus, fusiform gyrus and lingual gyrus); Braak IV (insula, inferior temporal, lateral temporal, posterior cingulate and inferior parietal); Braak V (orbitofrontal, superior temporal, inferior frontal, cuneus, anterior cingulate, supramarginal gyrus, lateral occipital, precuneus, superior parietal, superior frontal and rostromedial frontal) and Braak VI (pericentral and pericalcarine).

Second, to understand the relationship between inflammation, Aβ and tau, ^11^C-ER176, ^18^F-florbetaben and ^18^F-flortaucipir scans were segmented using a modified version of the Hammers’ probabilistic brain atlas,^49^ which provides a more anatomically detailed parcellation of the whole brain than the Braak regions. The original Hammers’ atlas includes 83 brain regions. However, some of the original regions were merged to avoid including very small regions that could lead to error in the analysis of PET data, with a relatively large FWHM. We merged the three different anterior temporal lobe regions from the Hammers’ atlas to one, named anterior temporal pole (ATP). Parahippocampal and fusiform gyri were also merged (PhF). Finally, the five different orbitofrontal gyri from the Hammers’ atlas were also merged to one, named orbitofrontal cortex (OFC). This modified version of the Hammers’ atlas with 71 regions was used to screen for tracer differences between patients and controls across the entire brain. For statistical comparisons and considering the neurobiology of AD in EOAD, all non-cortical regions except amygdala and hippocampus were excluded from analysis, giving a total of 46 regions compared across controls and patients. In an exploratory analysis, prompted by the well-known Aβ pathology in the caudate of patients with EOAD,^50^ inflammation, volume, Aβ and tau in the caudate nuclei were compared between MCI-EOAD and CU participants independently from the previous ROI analysis.

#### ^11^C-ER176 PET acquisition and processing

Twenty-five MCI-EOAD patients and 23 CU controls had ^11^C-ER176 PET scans. Dynamic images were acquired starting immediately after an automatic pump bolus injection of ∼20 mCi ^11^C-ER176. Images were acquired for 90 min and binned in 27 frames (6 frames × 0.5 min, 3 frames × 1 min, 2 frames × 2 min, 16 frames × 5 min). Radial arterial blood samples were drawn manually at 15-s intervals for the first 2.5 min, then at 3, 4, 5, 6, 8, 10, 15, 20, 30, 40, 50, 60, 75 and 90 min. Radioactivity in whole blood and plasma was measured by a gamma counter, and the parent concentration was obtained by high-performance liquid chromatography from 10 plasma samples at 5, 10, 15, 20, 30, 40, 50, 60, 75 and 90 min. The measured fractions of the parent were fit to an extended Hill function^51^ via the curve fitting toolbox of MATLAB (v.2023a, MathWorks, Natick, MA). Measured plasma activity was then multiplied by the fit parent fraction to obtain the metabolite-corrected arterial input function (AIF). The metabolite-corrected AIF was fit to a tri-exponential function for use in kinetic modelling. Free fraction in plasma (*f*_P_) was measured in duplicate for each participant by an ultrafiltration technique and normalized by a common standard.^52^ Since *f*_P_ was similar in controls (0.046 ± 0.014) and patients (0.052 ± 0.015), ^11^C-ER176 uptake was quantified using total distribution volume (*V*_T_) as the primary outcome measure.

At the voxel level, *V*_T_ parametric images were calculated using the AIF with the Logan plot method implemented in the PXMOD module of PMOD. Voxel-wise partial volume corrected (PVC) images were obtained using the voxel-wise Geometric Transfer Method (GTM) implemented in the PNEURO pipeline of PMOD 3.9, by using all segmented brain regions and the background activity. Using the PETsurfer pipeline implemented in Freesurfer 7,^53^ PVC corrected PET images were sampled onto the Freesurfer cortical surface of each subject. Individual surface maps were smoothed geodesically with FWHM of 8 mm and resampled onto the fsaverage template.

At the regional level, PET time-activity curves (TACs) for Tau-PET Braak ROIs were extracted and corrected for partial volume effect using the symmetric GTM method via the PETSurfer tools.^54^ TACs for the modified Hammers’ atlas were extracted and corrected for partial volume effect as described above.^55^*V*_T_ values were calculated using the AIF and the regional PVC corrected TACs with the Logan plot method. Kinetic modelling was performed using in-house MATLAB code available on our lab’s GitHub page.

#### Amyloid-β PET acquisition and processing

Twenty-three MCI-EOAD patients and 19 CU controls had Aβ PET scans using the tracers ^18^F-florbetaben (*n* = 40), ^11^C-PIB (*n* = 1) and ^18^F-florbetapir (*n* = 1). The ^18^F-florbetapir scan was performed for clinical use, offsite, on a different scanner. Owing to the relatively low quality of this scan, it was not included in correlation analyses and only used for assessing the Aβ status of the patient. ^18^F-florbetaben scans were acquired from 90 to 110 min in 4 × 5 min frames after a bolus injection of 8.1 ± 10% mCi, and the ^11^C-PIB scan was acquired from 50 to 70 min in 4 × 5 min frames after a bolus injection of 21.3 mCi.^56,57^ The four frames were averaged using PMOD. Standardized uptake value ratio (SUVR) values at the voxel and regional level were calculated from concentration values using the cerebellar grey matter as the reference region.

For voxelwise analyses, PVC corrected PET images were sampled onto the Freesurfer cortical surface of each subject and re-sampled onto the fsaverage template. Regional concentration values were calculated for the modified Hammers’ atlas using the same procedures as delineated above for ^11^C-ER176 TAC extraction.

Centiloids for the MCI-EOAD patients were calculated following published methods^56,57^; all patients with Aβ PET scans were determined to be Aβ-positive with a centiloid value of at least 28.^58^

#### ^18^F-flortaucipir PET acquisition and processing

Twenty-one MCI-EOAD patients and 23 CU controls had ^18^F-flortaucipir PET scans. ^18^F-flortaucipir images were acquired after a bolus injection of 10 ± 10% mCi from 80 to 100 min in 4 × 5 min frames. The four frames were averaged using PMOD. SUVR values were calculated from concentration values using the cerebellar grey matter as the reference region. SUVR images were processed at the voxel level identically to Aβ images described above to obtain PVC corrected images resampled onto fsaverage. Regional concentration values were calculated for both atlases using the same procedures as delineated above for ^11^C-ER176 TAC extraction.

### Statistical analysis

Surface-based analyses^59^ were performed to compare ^11^C-ER176, ^18^F-florbetaben and ^18^F-flortaucipir uptake in MCI-EOAD patients versus CU participants. Parametric images of the three tracers were analysed using a vertex-wise ANOVA for the effect of clinical status (MCI-EOAD versus CU). For ^11^C-ER176, *TSPO* genotype status was used as a covariate. Similarly, differences in cortical thickness between MCI-EOAD and CU participants were calculated using an ANOVA for the effect of clinical status. Statistical significance was corrected for multiple comparisons using the false discovery rate (FDR) functionality provided by Freesurfer.

To illustrate the brain topography of Aβ and tau in the sample of 25 patients with MCI-EOAD, vertex-wise frequency maps were created showing areas of the cerebral cortex with elevated ^18^F-florbetaben and ^18^F-flortaucipir uptake. MCI-EOAD patients’ surface SUVR values were thresholded at each vertex of the fsaverage surface. Frequency maps depict in each vertex the percentage of patients with uptake greater than the threshold value. Conservative thresholds were chosen: 1.5 SUVR for ^18^F-florbetaben (corresponding to a centiloid value of 75, https://www.gaain.org/centiloid-project) and 2.2 SUVR for ^18^F-flortaucipir.^60^

Region-wise analyses were performed to compare ^11^C-ER176 and ^18^F-flortaucipir uptake between MCI-EOAD and CU participants in the Tau-PET Braak-stage regions. Similarly, region-wise analyses were performed to compare ^11^C-ER176, ^18^F-florbetaben and ^18^F-flortaucipir uptake between MCI-EOAD and CU participants in the modified Hammers’ atlas. For both analyses, ANOVAs were calculated for the effect of clinical status (MCI-EOAD versus CU) to determine significant regional differences. *TSPO* genotype status was included as a covariate for ^11^C-ER176 analysis. Region-wise analyses were corrected using the Holm–Bonferroni procedure with a family-wise error (FWE) threshold of *p*_FWE_ < 0.05. Effect size (partial eta squared) and observed power were also calculated.

To determine inflammation, Aβ and tau in the caudate nucleus, multivariate analyses of variance (MANOVA) were calculated with group status (MCI-EOAD versus CU) as between-subject factor and right and left caudate as within-subject factor for each tracer. *TSPO* genotype was entered as a covariate for ^11^C-ER176. In the presence of significant main effects, follow-up univariate ANOVAs were examined. Effect size (partial eta squared) and observed power were also calculated.

Finally, regional volume was quantified using the PNEURO utility of PMOD. The volume of each region of interest in the modified Hammers’ atlas was calculated for the MCI-EOAD and CU participants who underwent ^11^C-ER176 and then normalized by the total intracranial volume as calculated by Freesurfer. To obtain a normalized measure of volume loss, ROI volumes for CU participants were used to generate *z*-scores of the patients’ volumes for each region of the modified Hammers’ atlas.

#### Inflammation, atrophy, amyloid-β and tau correlations

The regional co-localization among brain inflammation, Aβ and tau, as well as their relationship with atrophy in MCI-EOAD was compared in 21 of 25 MCI-EOAD participants scanned with all three PET tracers. Two distinct correlation analyses were conducted, namely inter-subject and intra-subject analyses.

First, to assess regional co-localization at the group level, inter-subject regional analyses were performed. Since the presence of three distinct TSPO affinities for ^11^C-ER176 precludes calculating average regional inflammation values for the entire sample, for each TSPO affinity group we calculated averages of inflammation *V*_T_, Aβ and tau SUVR, and volumetric *z*-scores for each of the Hammers’ atlas cortical regions. Pearson correlations of the average values were calculated between inflammation and Aβ, inflammation and tau, and Aβ and tau as well as all three PET tracers with atrophy. Standardized data were shown with a linear fit to graphically represent these correlations.

Second, while in AD both Aβ and tau localize mostly to brain structures of the default network, the areas affected can vary markedly across subjects.^61^ To account for this variability, an intra-subject correlation method was implemented, precluding the need to use the *TSPO* genotype as a covariate, and allowing us to consider the differences in tracer relationships within each subject for all three PET tracers. Using inflammation *V*_T_, Aβ and tau SUVR and volumetric *z*-scores in the ROIs of the modified Hammers’ atlas, individual Pearson correlations were calculated between inflammation and Aβ, inflammation and tau, and Aβ and tau, as well as all three PET tracers with atrophy. The significance of each correlation was assessed at *P* < 0.05 with FWE correction applied via the Holm–Bonferroni procedure. Correlation coefficients were averaged across all individuals and standard deviations were calculated.

To determine statistical significance of the pairwise correlations among inflammation, Aβ and tau, correlation values were normalized via the Fisher transformation and then compared via a paired *t-*test. A similar method was applied to the correlations between inflammation, Aβ and tau with volume. Significance of pairwise differences between correlations were assessed at *P* < 0.05 with FWE correction applied via the Holm–Bonferroni procedure.

#### Inflammation and neuropsychological test score correlations

To assess whether localized inflammation predicts neuropsychological decline, for each region of the modified Hammers’ atlas, we calculated a Pearson partial correlation between ^11^C-ER176 *V*_T_ values and MMSE score, DemTect total score, as well as the immediate and delayed recall sub-scores of the DemTect, with TSPO affinity as a covariate.

## Results

MCI-EOAD and CU controls in the ^11^C-ER176 cohort did not differ in sex (as assessed by a chi-squared test) or years of education (as assessed by a *t*-test). CU controls were slightly older than MCI-EOAD patients; however, this difference would be expected to blunt inflammation values in patients, since TSPO PET uptake increases with age.^9^ On neuropsychological evaluation, MCI-EOAD patients were significantly cognitively impaired across all domains used to screen for dementia, with the exception of language (Table 1), as well as on multiple domains in the more extended neuropsychological assessment battery from the NACC UDS v3.0, consisting of measures of attention, processing speed, executive function, episodic memory and language (Supplementary Table 1).

### Inflammation, atrophy, amyloid-β and tau in MCI-EOAD

**Figure 1:**
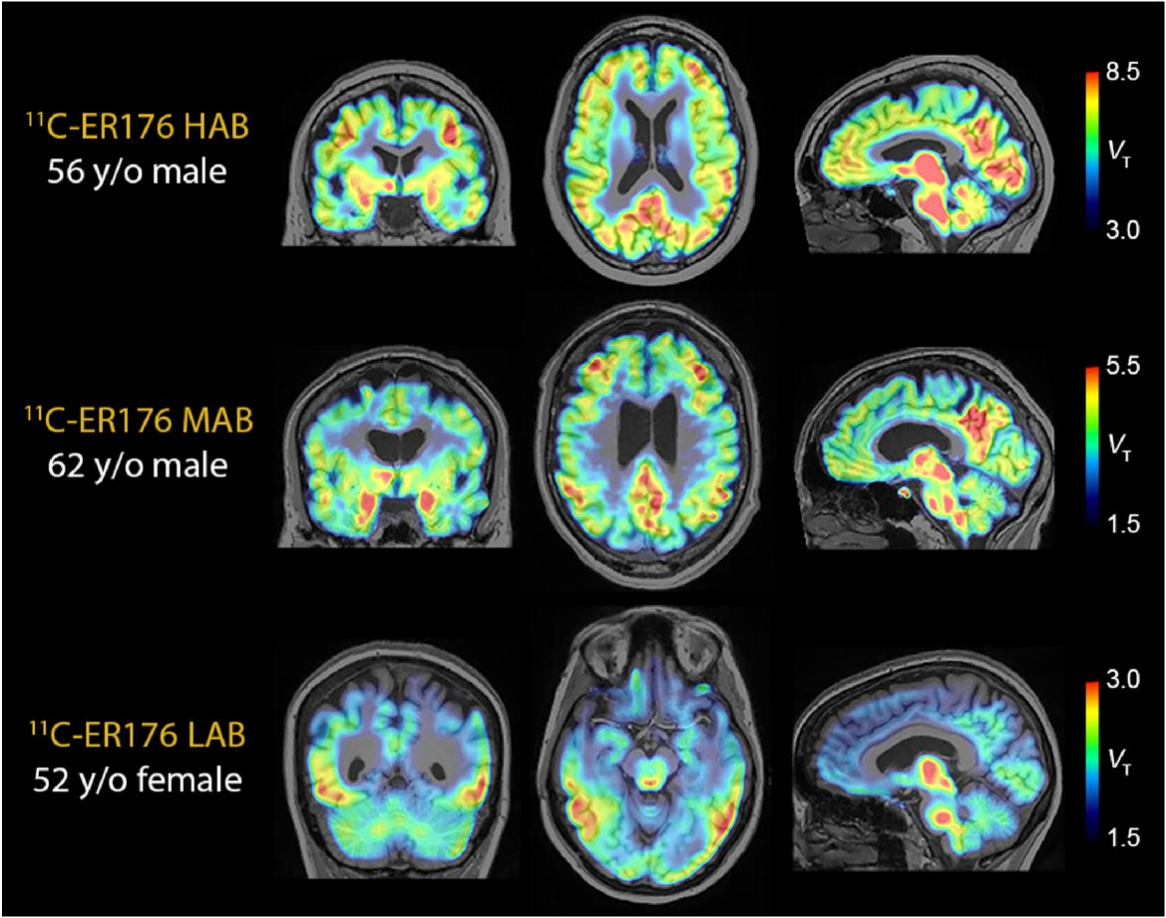
**^11^C-ER176 PET in all three genotypes.** Examples of ^11^C-ER176 signals in high- (HAB), mixed- (MAB) and low-affinity (LAB) binders from our mild cognitive impairment group. Bars on the *right* provide the thresholds at which total distribution volume (*V*_T_) values for each image are shown. The low-affinity binder had posterior cortical atrophy, with greatest involvement of posterior temporal cortex. The uptake in thalamus and brainstem is also present in normal controls. TSPO = translocator protein; y/o = years old.

**Figure 2:**
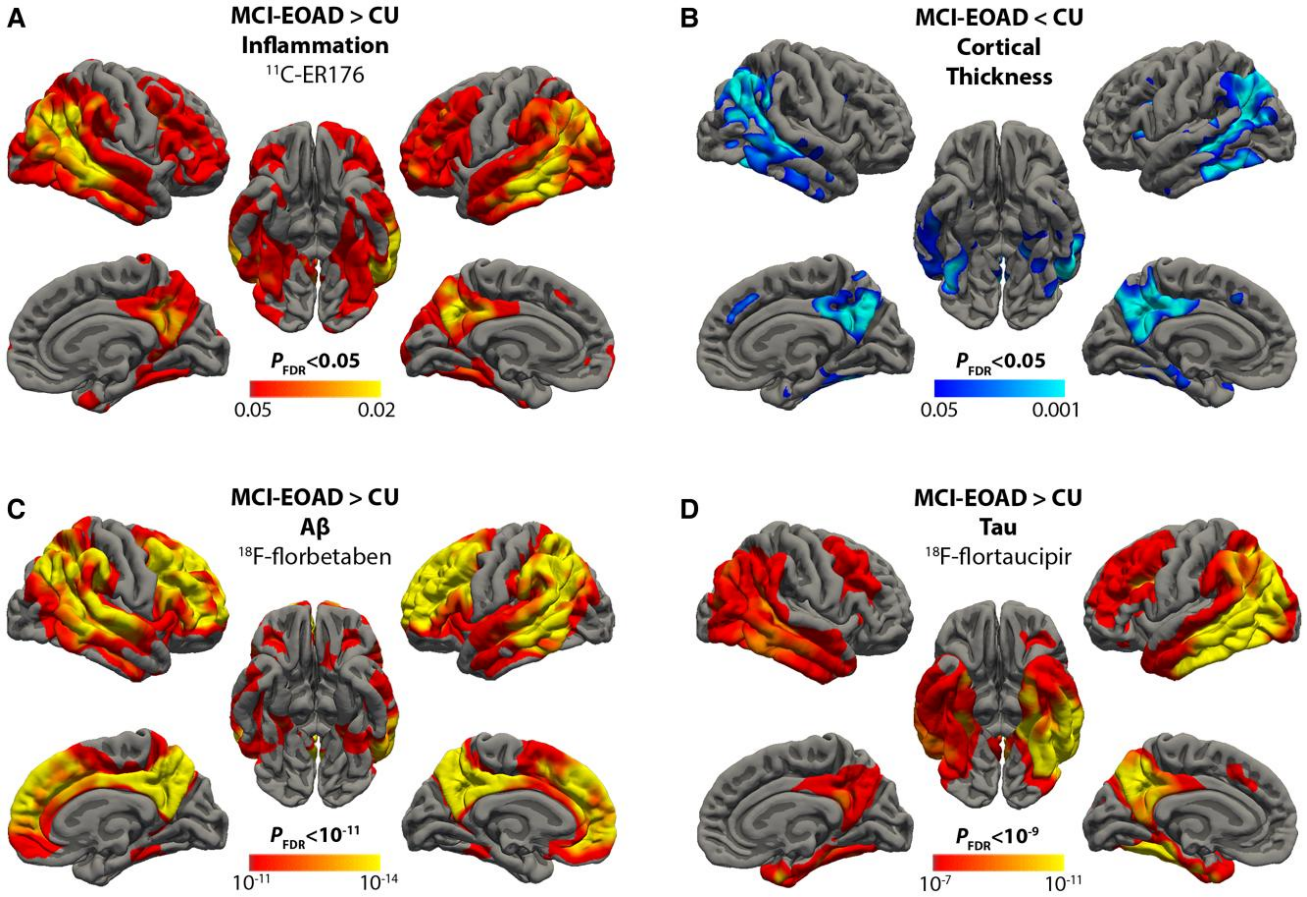
**Inflammation, cortical thickness, amyloid-β and tau in mild cognitive impairment early-onset Alzheimer’s disease (MCI-EOAD)**. (**A**) Surface-based ANCOVA comparing ^11^C-ER176 uptake in 25 MCI-EOAD patients and 23 cognitively unimpaired (CU) controls. Coloured vertices on the cortical surface map indicate areas where MCI-EOAD patients had greater inflammation than controls. (**B**) Cortical thickness maps comparing 25 MCI-EOAD subjects and 23 CU controls. Coloured vertices indicate areas where patients showed greater cortical atrophy than controls. (**C**) Surface-based ANOVA comparing ^18^F-florbetaben uptake in 21 MCI-EOAD patients and 19 CU controls. Coloured vertices on the cortical surface map indicate areas where MCI-EOAD patients had greater amyloid-β (Aβ) than controls. (**D**) Surface-based ANOVA comparing ^18^F-flortaucipir uptake in 21 MCI-EOAD patients and 23 CU controls. Coloured vertices on the cortical surface map indicate areas where MCI-EOAD patients had greater tau than controls. FDR = false discovery rate.

Using ^11^C-ER176 PET, we were able to image participants with any *TSPO* genotype and did not have to exclude or screen for enrolment based on binding affinity (Fig. 1). As expected, *V*_T_ differed across the three TSPO affinity groups (Supplementary Fig. 1). On surface-based analyses, inflammation measured with ^11^C-ER176 was elevated in MCI-EOAD, specifically in the classical Alzheimer’s cortical areas,^13^ many overlapping with the default mode network^62^ (Fig. 2A). In the temporal lobe, the middle and inferior temporal gyri were particularly affected, with relative preservation of the superior temporal gyrus. Vertex-based cortical thickness group comparisons showed atrophy of the well-known AD regions^63^ in the EOAD group (Fig. 2B). Surface-based group analysis and the vertex-wise frequency map showed elevated Aβ deposition in regions comprising the default network but with more involvement of the frontal lobe than for cortical atrophy (Fig. 2C and Supplementary Fig. 2A). The tau surface-based group analysis (Fig. 2D) and frequency map (Supplementary Fig. 2B) most closely resembled the topography of inflammation and, particularly, cortical atrophy (Fig. 2A and B). Similar topographic findings were derived from surface-based group analyses for the three different PET tracers when the analyses were not adjusted for partial volume effect (Supplementary Fig. 3).

**Figure 3:**
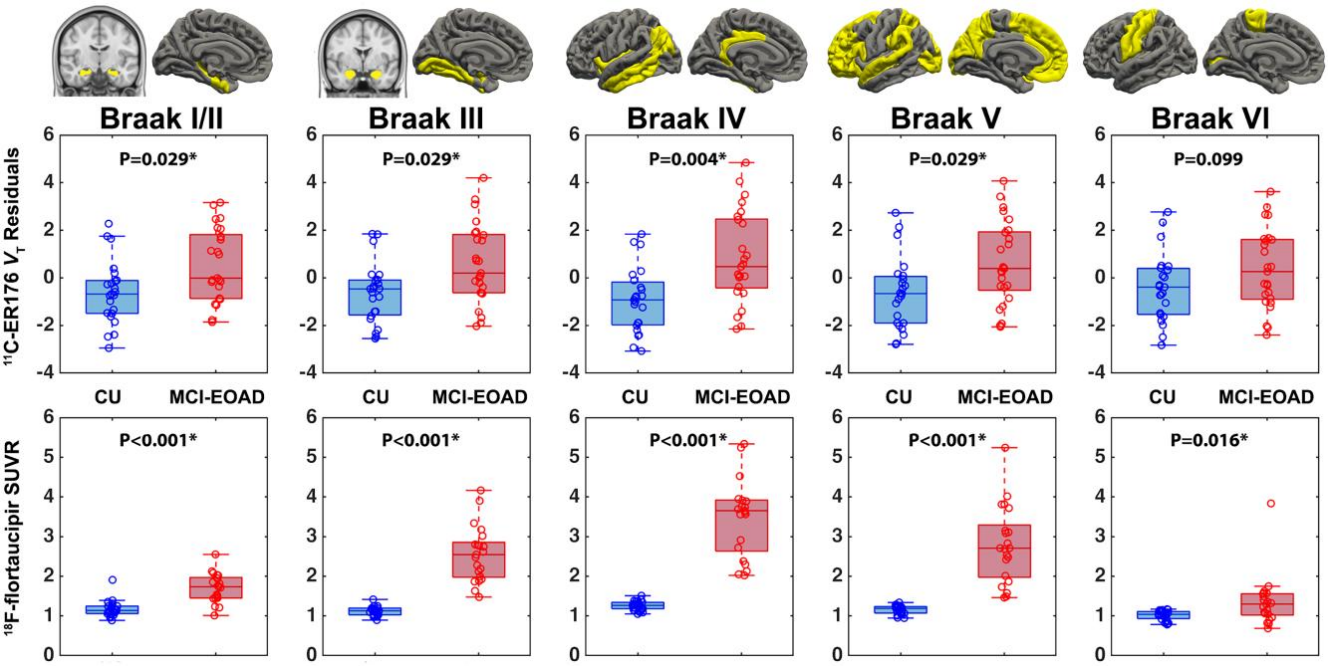
**Inflammation and tau in the Tau-PET Braak-stage regions**. In tau-PET Braak regions, modified from Pascoal *et al.*,^48^ box and whisker plots show values of ^11^C-ER176 (*top*) and ^18^F-flortaucipir (*bottom*) uptake in cognitively unimpaired (CU) controls and mild cognitive impairment early-onset Alzheimer’s disease (MCI-EOAD). *P*-values for group comparisons are adjusted using family-wise error (FWE) correction. SUVR = standardized uptake value ratio; *V*_T_ = total distribution volume.

The same regions shown to have increased inflammation by the more anatomically granular vertex-wise analysis (Fig. 2A) were shown to have significantly increased inflammation when partitioned according to Tau-PET Braak staging, even after correction for multiple comparisons (Fig. 3). Tau was widespread in the brain of MCI-EOAD patients, with the highest effect size in Braak IV region, *F*(1,42) = 98.71, *P* < 0.001 uncorrected, eta^2^ = 0.70, observed power = 1.00; ^11^C-ER176 uptake showed a similar pattern to tau accumulation across Braak stages, with the highest effect size also in Braak IV region, *F*(1,44) = 13.01, *P* < 0.001 uncorrected, eta^2^ = 0.23, observed power = 0.94. Unlike tau, in Tau-PET Braak VI region, ^11^C-ER176 was not statistically significantly increased in MCI-EOAD, *F*(1,44) = 2.85, *P* = 0.099 uncorrected, eta^2^ = 0.06, observed power = 0.38. Similar results were observed when the inflammation and tau regional group analyses were conducted without correcting the data for partial volume effect (Supplementary Fig. 4).

Regarding inflammation in the regions of the more anatomically detailed Hammers’s probabilistic atlas, 13 regions had increased inflammation in MCI-EOAD that was statistically significant even after correction for multiple comparisons (Supplementary Fig. 5). Those in both hemispheres included the middle frontal gyrus, supramarginal gyrus, precuneus, middle and inferior temporal gyri and posterior temporal region. The left posterior cingulate region, left lateral occipital gyri and right amygdala also had statistically significant increased inflammation. Without correcting for partial volume effect, a similar pattern was seen but did not survive FWE correction. To provide less restricted data, the results of region-wise ANOVAs, uncorrected for multiple comparisons, for inflammation ^11^C-ER176 *V*_T_ and tau ^18^F-flortaucipir SUVR are shown in Supplementary Table 3, with and without correction for partial volume effect. Both tracers had increased uptake (uncorrected *P* < 0.05) across the entire cortex, hippocampus and amygdala. For inflammation PET, the difference did not reach uncorrected *P* < 0.05 in the precentral cortex, subgenual, presubgenual and subcallosal cingulate bilaterally, and the right anterior cingulate. For tau PET, the difference did not reach uncorrected *P* < 0.05 in only the subgenual, presubgenual and subcallosal cingulate region bilaterally. Similar statistical significance patterns of global uptake were observed for both PETs, inflammation and tau, when not accounting for partial volume effect with only minor exceptions: inflammation PET did also not reach statistical significance in the left anterior cingulum and the left postcentral cortex, while tau PET exhibited statistical significance bilaterally in the subgenual, presubgenual and subcallosal cingulate region.

The caudate nucleus, which had marked Aβ deposition in this sample [*F*(2,37) = 41.62, *P* < 0.001 uncorrected, eta^2^ = 0.69, observed power = 1.00], showed to a much lesser extent increased inflammation [*F*(2,43) = 3.79, *P* = 0.030 uncorrected, eta^2^ = 0.15, observed power = 0.66] and tau [*F*(2,41) = 9.60, *P* < 0.001 uncorrected, eta^2^ = 0.32, observed power = 0.97] (Supplementary Fig. 6).

### Inflammation, atrophy, amyloid-β and tau: correlations of their brain locations in MCI-EOAD

**Figure 4:**
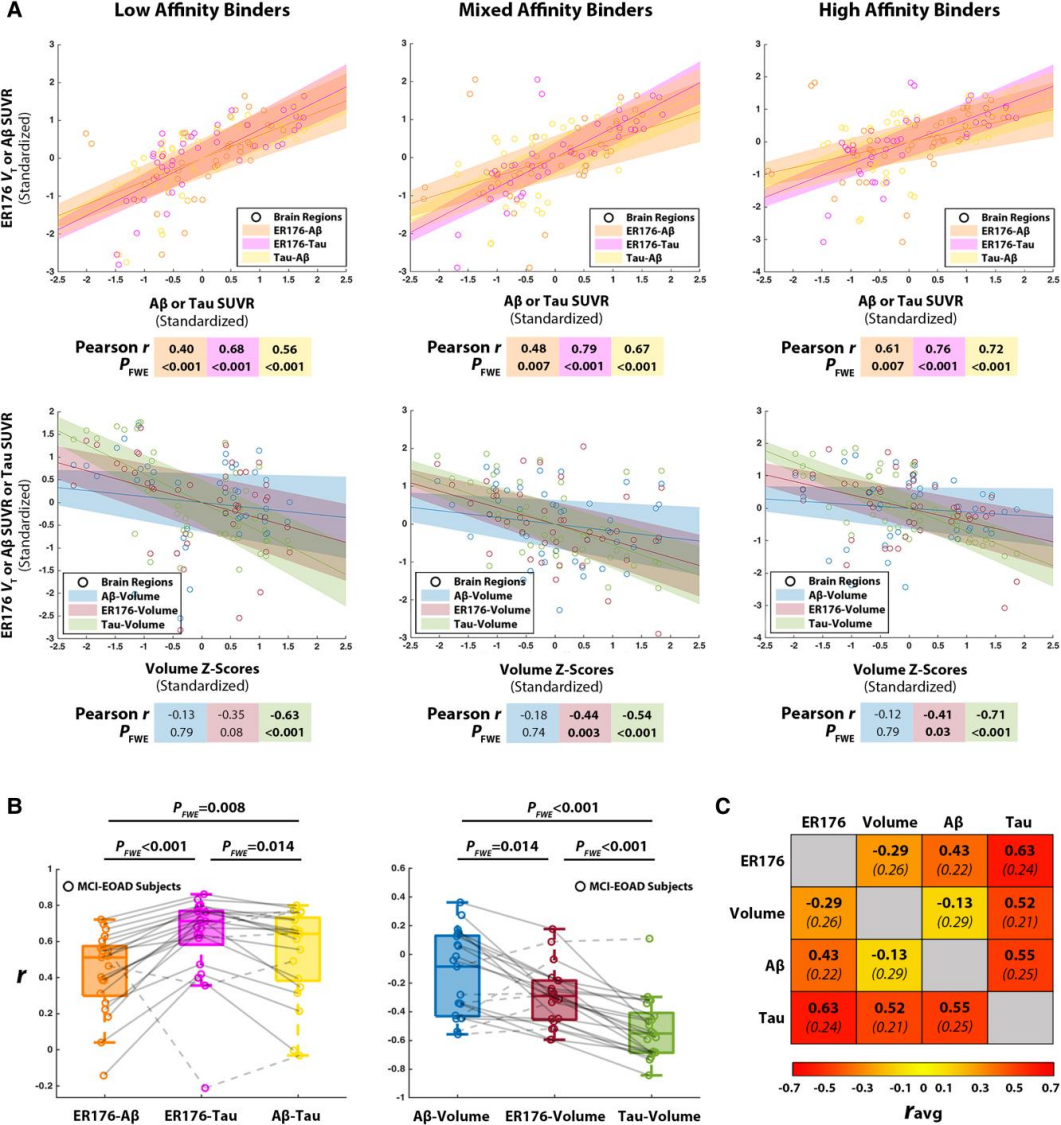
**Correlations across regional inflammation, cortical thickness, amyloid-β and tau**. (**A**) Scatter plots showing the pairwise relationships between inflammation, amyloid-β (Aβ) and tau PETs (*top*), as well as each PET tracer’s relationships with volume (*bottom*) across all three TSPO affinity groups. Data are standardized so that regression slopes equal correlation coefficients. Inflammation and tau exhibit the strongest of all pairwise correlations within each affinity. (**B**) Box plots display Pearson correlation values for each pairwise PET correlation as well as each PET tracer’s correlation with volume on an individual subject basis. *P*-values for comparisons are family-wise error (FWE)-corrected. (**C**) The matrix indicates the average, denoted as *r*_avg_, of all single Pearson intra-individual correlation values in Mild Cognitive Impairment caused by early-onset Alzheimer’s disease (MCI-EOAD) subjects. Among individual FWE-corrected correlations, inflammation and tau were significant in 16/21 subjects, Aβ and tau in 15/21, inflammation and Aβ in 11/22, tau and volume in 13/21, inflammation and volume in 7/25 and Aβ and volume in 2/22. ER176 = ^11^C-ER176 inflammation PET; SUVR = standardized uptake value ratio.

Inter-subject regional correlations for each of the three TSPO affinities are shown in Fig. 4A. For all three affinities, the correlation between inflammation ^11^C-ER176 *V*_T_ and tau ^18^F-flortaucipir SUVR was highest of all the correlations among the PET tracers or with volume.

Intra-subject correlations for each MCI-EOAD participant across all cortical regions plus amygdala and hippocampus, between ^11^C-ER176 *V*_T_, ^18^F-florbetaben SUVR, ^18^F-flortaucipir and volume *z*-scores are presented in Supplementary Fig. 7A andB, respectively, with and without partial volume effect correction. A matrix of averages of intra-subject correlations between inflammation, atrophy, Aβ and tau is presented in Fig. 4C. Of the three average PET tracer correlations calculated (inflammation and tau; inflammation and Aβ; and tau and Aβ), the average correlation between inflammation and tau (*r* = 0.63 ± 0.24) was highest, followed by Aβ and tau (*r* = 0.55± 0.25) and inflammation and Aβ (*r* = 0.43 ± 0.22). PET tracer uptakes correlated also with MRI cortical volume (Fig. 4C). Highest was the correlation between tau and volume (*r* = −0.52 ± 0.21), followed by inflammation and volume (*r* = −0.29 ± 0.26) and Aβ and volume (*r* = −0.13 ± 0.29). Across subjects, all significant correlations were negative, except for one subject with a positive Aβ versus volume correlation and one subject with a positive inflammation versus volume correlation.

Inflammation–tau correlations were statistically significantly stronger than Aβ–tau and inflammation–Aβ correlations (Fig. 4B). Tau–volume correlations were stronger than inflammation–atrophy, and inflammation–volume correlations surpassed Aβ–volume correlations (Fig. 4B).

### Inflammation and neuropsychological test score correlations

**Figure 5:**
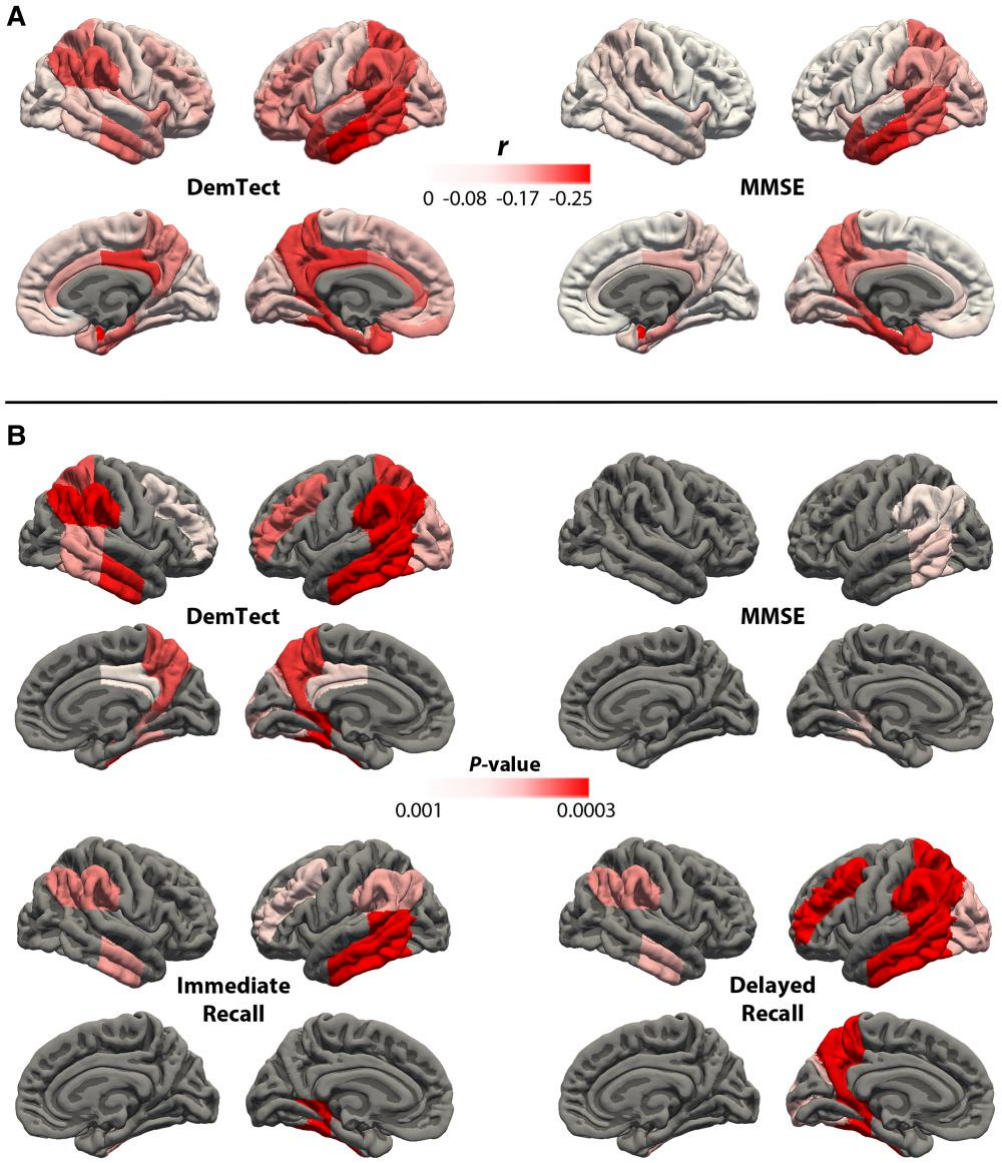
**Correlation between the topography of ^11^C-ER176 PET inflammation uptake and neuropsychological scores.** (**A**) Maps show the Pearson correlation coefficients between inflammation in Hammers’ atlas regions and cognitive scores from the DemTect and Mini-Mental Status Examination (MMSE) in the mild cognitive impairment caused by early-onset Alzheimer’s disease (MCI-EOAD) sample, not including cognitively unimpaired (CU) controls. No correlations were statistically significant. (**B**) Maps show uncorrected *P*-values for Hammer’s atlas regions with statistically significant Pearson correlations (*P* < 0.001) between inflammation and the neuropsychological scores on DemTect, MMSE and immediate and delayed recall sub-tests of the DemTect in the entire sample, including patients and controls. FWE = family-wise error.

In our group of 48 participants (25 MCI-EOAD and 23 CU), ^11^C-ER176 uptake significantly correlated with MMSE score, total DemTect score and the immediate and delayed recall sub-scores of the DemTect (Fig. 5). Correlations were strongest with the DemTect and the correlated brain regions corresponded to those known to be affected in AD (Fig. 5B). Since our patients with MCI-EOAD were at a similar level of impairment (CDR of 0.5), we did not find statistically significant correlations when excluding CU participants. However, within the MCI-EOAD group and excluding CU participants, the same anatomic pattern was found between inflammation and the MMSE and DemTect scores (Fig. 5A).

## Discussion

Our study demonstrated the high anatomic specificity of inflammation in MCI-EOAD (Fig. 2). Most affected areas corresponded to postcentral components of the default network,^64^ tracking well the Alzheimer’s brain topography signature.^63,65^ In addition, our study showed for the first time the usefulness of ^11^C-ER176 to measure robustly brain inflammation in AD. ^11^C-ER176 facilitates the study of inflammation, since it precludes the need to screen for enrolment based on binding affinity and to exclude low-affinity binders (Figs 1 and 4). Finally, our study showed that the brain localization of inflammation had a closer correlation with the localization of tau than with that of Aβ, and this correlation was even higher than the correlation between Aβ and tau brain topography.

Our patient sample had the advantage of being quite homogeneous, composed only of EOAD, likely to harbour few co-pathologies,^11,12^ and with a CDR = 0.5 and therefore in the MCI cognitive stage. However, as shown for other EOAD samples,^13,61^ imaging tau staging showed most of our patients to be at the Braak V stage^66^ (Fig. 3), thus having already advanced tau pathology despite their relatively spared cognitive abilities. The brain topography of both Aβ and tau (Fig. 2C and D) in our sample, which was drawn from the LEADS study,^41^ reproduced closely similar topography in the entire baseline EOAD LEADS cohort, composed of 243 EOAD patients.^13^

Although to limit participant radiation exposure, we did not compare head-to-head two TSPO PET tracers, as we have done in previous studies,^33,67^ regions with significantly increased ^11^C-ER176 signal compared favourably with those reported in AD using other recent tracers.^68,69,70,71^ As with other TSPO tracers, including ^11^-C-PK11195,^26^ binding was influenced by the *TSPO* polymorphism. However, the high binding affinity of ^11^C-ER176 allowed us to study particularly well the topography of inflammation in EOAD, as well as its relationship with the topography of volume loss, Aβ and tau in the brain.

Mostly from rodent data, the TSPO signal has been thought to reflect ‘activated’ microglia,^28,72^ a construct widely used in neuropathology.^73^ Recent data suggest that, in humans, TSPO PET does not reflect ‘activation’ but rather the regional density of microglia and migrated macrophages, both critical components of the brain inflammatory response.^21^ The striking topographic selectivity of increased TSPO signal in our study, involving particularly the association cortex of parietal and temporal lobes, while sparing primary cortex (sensory-motor and visual, Fig. 2; primary auditory, not shown) highlights the usefulness of TSPO PET to study a neurobiologically relevant component of AD, in this case inflammation. Our study indicates that regions of the brain known to be selectively involved by the AD process^74^ have increased inflammation, which, at the cellular level, may reflect microglial proliferation, macrophage migration from the blood and, possibly, changes in astrocytes and endothelial cells,^22^ all of which are neuropathologically prominent in AD-involved regions but with regional variability, as are Aβ and tau density.^73^

Unlike in a study of semantic dementia, where inflammation peaked at the margin between thinned and volume-normal cortex, perhaps suggesting a role for inflammation in damage propagation,^75^ in this MCI-EOAD sample, inflammation peaked at the regions with most atrophy (Fig. 2A and B). Furthermore, in most subjects, there was a negative correlation between inflammation and cortical thickness. Remarkably, in one single subject, with an MMSE of 30 and therefore at a very early disease stage, the correlation was in the opposite direction, perhaps suggesting that increased inflammation may cause transient cortical swelling, as found at early stages of autosomal-dominant AD.^76^

In our study, inflammation correlated better with tau than with Aβ, as described at post-mortem^77^ and in previous TSPO PET studies, particularly those performed at more advanced stages of the disease.^8,9,28^ Our sample was at the imaging tau Braak stage V. At this stage, we found the typical partial disconnect between Aβ and tau topography.^13^ Aβ was markedly increased in the entire default and related networks,^64^ including frontal cortex (Fig. 2C) and in the caudate nucleus (Supplementary Fig. 6), which is positively connected to the cortical default network.^78^ In contrast, although tau was widely increased, including in the frontal lobes, the highest density was in post-Rolandic regions (Fig. 2D), with smaller effect sizes in frontal lobes (Fig. 2D) and caudate nucleus (Supplementary Fig. 6). It is likely that these different brain regions have different susceptibilities to either Aβ deposition or to the neuronal changes leading to tau buildup.^79^ Different susceptibilities might be phylogenetic in origin, as the post-Rolandic component of the default network, where tau tends to accumulate, is more closely shared by humans and non-human primates than the anterior component.^80,81^ Not only topography differs. While Aβ aggregates, as detectable by current PET tracers, seem to have widespread simultaneous accumulation throughout the brain,^82,83^ tau spreads over years along natural brain networks.^84,85^ Another factor to consider when trying to explain the relative disconnect between the anatomic distribution of Aβ and tau at this stage of AD is the role of microglia.

The close topographic relationship of inflammation to tau in our study (Fig. 2) supports the concept that microglia are closely associated with tau build-up.^28^ In experimental animal models, microglia contribute to tau spreading from sick to healthy neurons,^86^ particularly in the presence of increased Aβ,^87^ a well-known risk for tau spreading.^88,89^ A microglial role in humans is suggested by the association of tau with increased inflammation documented in this and previous PET studies.^8,9,28,68^ Since microglia structure and function vary across brain regions, even in the healthy brain,^90^ some of the topographic distribution of tau might be related to topographic microglia variability.

### Limitations

If increased inflammation predisposes to tau build-up, brain regions may be postulated that have increased inflammation but where tau is not yet present. However, our study lacked longitudinal data, and in our cross-sectional study, we did not find regions where inflammation was significantly increased but tau was normal, which might have suggested that inflammation predisposes to subsequent tau build-up. It is possible that this potential finding was masked by TSPO imaging having greater variability among CU controls than tau imaging (Fig. 3), thus blunting TSPO signal significance in MCI-EOAD.

The lack of cellular specificity of TSPO, which is expressed not only by microglia but also by peripherally derived macrophages, astrocytes and endothelial cells, prevented us from defining the topography and association with Aβ and tau of each of these cell types. However, as in AD, their topographic increase is interrelated^91^; areas of brain inflammation may be easier to detect with a TSPO tracer than with a more selective microglial marker. For instance, while an abnormal signal was detected in the motor cortex of patients with amyotrophic lateral sclerosis using TSPO tracers,^24,92^ in the same patients, no signal was detected with a more cell-selective microglial purine receptor P2X7 tracer.^24^

Given that the TSPO tracer we used, ^11^C-ER176, is still novel and that the entire brain has TSPO expressing cells, we did not use a reference region to calculate BP_ND_ but instead used an arterial input function,^22^ which requires obtaining serial blood samples from a catheter in the radial artery, a procedure that is more cumbersome and invasive than used for reference-region based PET. Nonetheless, the cerebellum, with little or no inflammation, particularly at early AD stages, has been used or suggested as a reference region for TSPO PET in AD.^93-95^ Other suggested pseudo-reference regions have included the caudate nucleus^8^ and Braak region VI.^96^ Finally, the use of a PET tracer label with the ^11^C isotope requires the availability of a cyclotron at the site of use, because ^11^C has too short a half-life to be shipped. On the positive side, tracers labelled with ^11^C limit radiation exposure to the participants and allow for two different PET studies to be performed on the same day.

In summary, by using a sample with a purer form of AD and at a similar stage of the disease, our study provides statistically robust information on the topography of inflammation in human AD. Since the behaviour of microglia has been shown to differ greatly in humans as compared to rodent models of AD,^97^ our study contributes to the pool of information essential for the design and execution of AD clinical trials evaluating available medications that target brain inflammation.^98^

## Supplementary Material

awae234_Supplementary_Data

## Data Availability

Data that support the findings in this study are available from the corresponding author upon reasonable request.

## References

[1] Bettcher BM , Tansey MG, Dorothée G, Heneka MT. Peripheral and central immune system crosstalk in Alzheimer disease—A research prospectus. Nat Rev Neurol. 2021;17:689–701. 34522039 10.1038/s41582-021-00549-x PMC8439173

[2] Chen X , Firulyova M, Manis M, et al Microglia-mediated T cell infiltration drives neurodegeneration in tauopathy. Nature. 2023;615:668–677. 36890231 10.1038/s41586-023-05788-0 PMC10258627

[3] Lee CY , Landreth GE. The role of microglia in amyloid clearance from the AD brain. J Neural Transm (Vienna). 2010;117:949–960. 20552234 10.1007/s00702-010-0433-4 PMC3653296

[4] Odfalk KF , Bieniek KF, Hopp SC. Microglia: Friend and foe in tauopathy. Prog Neurobiol. 2022;216:102306. 35714860 10.1016/j.pneurobio.2022.102306 PMC9378545

[5] McAlpine CS , Park J, Griciuc A, et al Astrocytic interleukin-3 programs microglia and limits Alzheimer's disease. Nature. 2021;595:701–706. 34262178 10.1038/s41586-021-03734-6 PMC8934148

[6] Wang ZB , Ma YH, Sun Y, et al Interleukin-3 is associated with sTREM2 and mediates the correlation between amyloid-β and tau pathology in Alzheimer's disease. J Neuroinflammation. 2022;19:316. 36578067 10.1186/s12974-022-02679-5 PMC9798566

[7] Pelkmans W , Shekari M, Brugulat-Serrat A, et al Astrocyte biomarkers GFAP and YKL-40 mediate early Alzheimer's disease progression. Alzheimers Dement. 2024;20:483–493. 37690071 10.1002/alz.13450 PMC10917053

[8] Bradburn S , Murgatroyd C, Ray N. Neuroinflammation in mild cognitive impairment and Alzheimer's disease: A meta-analysis. Ageing Res Rev. 2019;50:1–8. 30610927 10.1016/j.arr.2019.01.002

[9] Masdeu JC , Pascual B, Fujita M. Imaging neuroinflammation in neurodegenerative disorders. J Nucl Med. 2022;63(Suppl 1):45S–52S. 35649654 10.2967/jnumed.121.263200

[10] Tanner JA , Iaccarino L, Edwards L, et al Amyloid, tau and metabolic PET correlates of cognition in early and late-onset Alzheimer's disease. Brain. 2022;145:4489–4505. 35762829 10.1093/brain/awac229 PMC10200306

[11] Spina S , La Joie R, Petersen C, et al Comorbid neuropathological diagnoses in early versus late-onset Alzheimer's disease. Brain. 2021;144:2186–2198. 33693619 10.1093/brain/awab099 PMC8502474

[12] Robinson JL , Lee EB, Xie SX, et al Neurodegenerative disease concomitant proteinopathies are prevalent, age-related and APOE4-associated. Brain. 2018;141:2181–2193. 29878075 10.1093/brain/awy146 PMC6022546

[13] Cho H , Mundada NS, Apostolova LG, et al Amyloid and tau-PET in early-onset AD: Baseline data from the Longitudinal Early-onset Alzheimer's Disease Study (LEADS). Alzheimers Dement. 2023;19 Suppl 9(Suppl 9):S98–S114. 37690109 10.1002/alz.13453 PMC10807231

[14] Fan Z , Brooks DJ, Okello A, Edison P. An early and late peak in microglial activation in Alzheimer's disease trajectory. Brain. 2017;140:792–803. 28122877 10.1093/brain/aww349 PMC5837520

[15] Ismail R , Parbo P, Madsen LS, et al The relationships between neuroinflammation, beta-amyloid and tau deposition in Alzheimer's disease: A longitudinal PET study. J Neuroinflammation. 2020;17:151. 32375809 10.1186/s12974-020-01820-6 PMC7203856

[16] Kreisl WC , Lyoo CH, McGwier M, et al In vivo radioligand binding to translocator protein correlates with severity of Alzheimer's disease. Brain. 2013;136(Pt 7):2228–2238. 23775979 10.1093/brain/awt145 PMC3692038

[17] Okello A , Edison P, Archer HA, et al Microglial activation and amyloid deposition in mild cognitive impairment: A PET study. Neurology. 2009;72:56–62. 19122031 10.1212/01.wnl.0000338622.27876.0d PMC2817573

[18] Parbo P , Ismail R, Sommerauer M, et al Does inflammation precede tau aggregation in early Alzheimer's disease? A PET study. Neurobiol Dis. 2018;117:211–216. 29902557 10.1016/j.nbd.2018.06.004

[19] Tondo G , Iaccarino L, Caminiti SP, et al The combined effects of microglia activation and brain glucose hypometabolism in early-onset Alzheimer's disease. Alzheimers Res Ther. 2020;12:50. 32354345 10.1186/s13195-020-00619-0 PMC7193377

[20] Zhang M , Qian XH, Hu J, et al Integrating TSPO PET imaging and transcriptomics to unveil the role of neuroinflammation and amyloid-beta deposition in Alzheimer's disease. Eur J Nucl Med Mol Imaging. 2023;51:455–467. 37801139 10.1007/s00259-023-06446-3 PMC10774172

[21] Nutma E , Fancy N, Weinert M, et al Translocator protein is a marker of activated microglia in rodent models but not human neurodegenerative diseases. Nat Commun. 2023;14:5247. 37640701 10.1038/s41467-023-40937-z PMC10462763

[22] Turkheimer FE , Rizzo G, Bloomfield PS, et al The methodology of TSPO imaging with positron emission tomography. Biochem Soc Trans. 2015;43:586–592. 26551697 10.1042/BST20150058 PMC4613512

[23] Ratai EM , Alshikho MJ, Zurcher NR, et al Integrated imaging of [(11)C]-PBR28 PET, MR diffusion and magnetic resonance spectroscopy (1)H-MRS in amyotrophic lateral sclerosis. Neuroimage Clin. 2018;20:357–364. 30112276 10.1016/j.nicl.2018.08.007 PMC6092554

[24] Van Weehaeghe D , Van Schoor E, De Vocht J, et al TSPO versus P2X7 as a target for neuroinflammation: An in vitro and in vivo study. J Nucl Med. 2020;61:604–607. 31562223 10.2967/jnumed.119.231985 PMC7198372

[25] Fujita M , Kobayashi M, Ikawa M, et al Comparison of four 11C-labeled PET ligands to quantify translocator protein 18 kDa (TSPO) in human brain: (R)-PK11195, PBR28, DPA-713, and ER176-based on recent publications that measured specific-to-non-displaceable ratios. EJNMMI Res. 2017;7(1):84. 29038960 10.1186/s13550-017-0334-8 PMC5643834

[26] Kreisl WC , Fujita M, Fujimura Y, et al Comparison of [11C]-(R)-PK 11195 and [11C]PBR28, two radioligands for translocator protein (18 kDa) in human and monkey: Implications for positron emission tomographic imaging of this inflammation biomarker. Neuroimage. 2010;49:2924–2932. 19948230 10.1016/j.neuroimage.2009.11.056 PMC2832854

[27] Owen DR , Guo Q, Kalk NJ, et al Determination of [(11)C]PBR28 binding potential in vivo: A first human TSPO blocking study. J Cereb Blood Flow Metab. 2014;34:989–994. 24643083 10.1038/jcbfm.2014.46 PMC4050243

[28] Pascoal TA , Benedet AL, Ashton NJ, et al Microglial activation and tau propagate jointly across Braak stages. Nat Med. 2021;27:1592–1599. 34446931 10.1038/s41591-021-01456-w

[29] Ferrari-Souza JP , Lussier FZ, Leffa DT, et al APOEε4 associates with microglial activation independently of Aβ plaques and tau tangles. Sci Adv. 2023;9:eade1474. 37018391 10.1126/sciadv.ade1474 PMC10075966

[30] Owen DR , Yeo AJ, Gunn RN, et al An 18-kDa translocator protein (TSPO) polymorphism explains differences in binding affinity of the PET radioligand PBR28. J Cereb Blood Flow Metab. 2012;32:1–5. 22008728 10.1038/jcbfm.2011.147 PMC3323305

[31] Zanotti-Fregonara P , Zhang Y, Jenko KJ, et al Synthesis and evaluation of translocator 18 kDa protein (TSPO) positron emission tomography (PET) radioligands with low binding sensitivity to human single nucleotide polymorphism rs6971. ACS Chem Neurosci. 2014;5:963–971. 25123416 10.1021/cn500138n PMC4210126

[32] Ikawa M , Lohith TG, Shrestha S, et al 11C-ER176, a radioligand for 18-kDa translocator protein, has adequate sensitivity to robustly image all three affinity genotypes in human brain. J Nucl Med. 2017;58:320–325. 27856631 10.2967/jnumed.116.178996 PMC5288742

[33] Zanotti-Fregonara P , Pascual B, Veronese M, et al Head-to-head comparison of (11)C-PBR28 and (11)C-ER176 for quantification of the translocator protein in the human brain. Eur J Nucl Med Mol Imaging. 2019;46:1822–1829. 31152207 10.1007/s00259-019-04349-w

[34] Paolicelli RC , Sierra A, Stevens B, et al Microglia states and nomenclature: A field at its crossroads. Neuron. 2022;110:3458–3483. 36327895 10.1016/j.neuron.2022.10.020 PMC9999291

[35] Morris JC . The Clinical Dementia Rating (CDR): Current version and scoring rules. Neurology. 1993;43:2412–2414. 10.1212/wnl.43.11.2412-a 8232972

[36] Folstein MF , Folstein SE, McHugh PR. “Mini-Mental State”: A practical method for grading the cognitive state of patients for the clinician. J Psychiatr Res. 1975;12:189–198. 1202204 10.1016/0022-3956(75)90026-6

[37] Kalbe E , Kessler J, Calabrese P, et al DemTect: A new, sensitive cognitive screening test to support the diagnosis of mild cognitive impairment and early dementia. Int J Geriatr Psychiatry. 2004;19:136–143. 14758579 10.1002/gps.1042

[38] Solomon PR , Hirschoff A, Kelly B, et al A 7 minute neurocognitive screening battery highly sensitive to Alzheimer's disease. Arch Neurol. 1998;55:349–355. 9520009 10.1001/archneur.55.3.349

[39] Mathuranath PS , Nestor PJ, Berrios GE, Rakowicz W, Hodges JR. A brief cognitive test battery to differentiate Alzheimer's disease and frontotemporal dementia. Neurology. 2000;55:1613–1620. 11113213 10.1212/01.wnl.0000434309.85312.19

[40] Weintraub S , Besser L, Dodge HH, et al Version 3 of the Alzheimer disease centers’ neuropsychological test battery in the Uniform Data Set (UDS). Alzheimer Dis Assoc Disord. 2018;32:10–17. 29240561 10.1097/WAD.0000000000000223 PMC5821520

[41] Apostolova LG , Aisen P, Eloyan A, et al The Longitudinal Early-onset Alzheimer's Disease Study (LEADS): Framework and methodology. Alzheimers Dement. 2021;17:2043–2055. 34018654 10.1002/alz.12350 PMC8939858

[42] Fischl B , Dale AM. Measuring the thickness of the human cerebral cortex from magnetic resonance images. Proc Natl Acad Sci U S A. 2000;97:11050–11055. 10984517 10.1073/pnas.200033797 PMC27146

[43] Fischl B , Sereno MI, Dale AM. Cortical surface-based analysis. II: Inflation, flattening, and a surface-based coordinate system. Neuroimage. 1999;9:195–207. 9931269 10.1006/nimg.1998.0396

[44] Joshi A , Koeppe RA, Fessler JA. Reducing between scanner differences in multi-center PET studies. Neuroimage. 2009;46:154–159. 19457369 10.1016/j.neuroimage.2009.01.057 PMC4308413

[45] Betthauser TJ , Bilgel M, Koscik RL, et al Multi-method investigation of factors influencing amyloid onset and impairment in three cohorts. Brain. 2022;145:4065–4079. 35856240 10.1093/brain/awac213 PMC9679170

[46] Weiner MW , Veitch DP, Aisen PS, et al Recent publications from the Alzheimer's Disease Neuroimaging Initiative: Reviewing progress toward improved AD clinical trials. Alzheimers Dement. 2017;13:e1–e85. 28342697 10.1016/j.jalz.2016.11.007 PMC6818723

[47] Macedo AC , Tissot C, Therriault J, et al The use of tau PET to stage Alzheimer disease according to the Braak staging framework. J Nucl Med. 2023;64:1171–1178. 37321820 10.2967/jnumed.122.265200 PMC10394315

[48] Pascoal TA , Therriault J, Benedet AL, et al 18F-MK-6240 PET for early and late detection of neurofibrillary tangles. Brain. 2020;143:2818–2830. 32671408 10.1093/brain/awaa180

[49] Hammers A , Allom R, Koepp MJ, et al Three-dimensional maximum probability atlas of the human brain, with particular reference to the temporal lobe. Hum Brain Mapp. 2003;19:224–247. 12874777 10.1002/hbm.10123 PMC6871794

[50] Kim JE , Lee DK, Hwang JH, et al Regional comparison of imaging biomarkers in the striatum between early- and late-onset Alzheimer's disease. Exp Neurobiol. 2022;31:401–408. 36631848 10.5607/en22022 PMC9841745

[51] Collste K , Forsberg A, Varrone A, et al Test-retest reproducibility of [(11)C]PBR28 binding to TSPO in healthy control subjects. Eur J Nucl Med Mol Imaging. 2016;43:173–183. 26293827 10.1007/s00259-015-3149-8

[52] Gandelman MS , Baldwin RM, Zoghbi SS, Zea-Ponce Y, Innis RB. Evaluation of ultrafiltration for the free-fraction determination of single photon emission computed tomography (SPECT) radiotracers: beta-CIT, IBF, and iomazenil. J Pharm Sci. 1994;83:1014–1019. 7965658 10.1002/jps.2600830718

[53] Greve DN , Svarer C, Fisher PM, et al Cortical surface-based analysis reduces bias and variance in kinetic modeling of brain PET data. Neuroimage. 2014;92:225–236. 24361666 10.1016/j.neuroimage.2013.12.021 PMC4008670

[54] Greve DN , Salat DH, Bowen SL, et al Different partial volume correction methods lead to different conclusions: An (18)F-FDG-PET study of aging. Neuroimage. 2016;132:334–343. 26915497 10.1016/j.neuroimage.2016.02.042 PMC4851886

[55] Thomas BA , Erlandsson K, Modat M, et al The importance of appropriate partial volume correction for PET quantification in Alzheimer's disease. Eur J Nucl Med Mol Imaging. 2011;38:1104–1119. 21336694 10.1007/s00259-011-1745-9

[56] Rowe CC , Doré V, Jones G, et al (18)F-florbetaben PET beta-amyloid binding expressed in Centiloids. Eur J Nucl Med Mol Imaging. 2017;44:2053–2059. 28643043 10.1007/s00259-017-3749-6 PMC5656696

[57] Klunk WE , Koeppe RA, Price JC, et al The Centiloid Project: Standardizing quantitative amyloid plaque estimation by PET. Alzheimers Dement. 2015;11:1–15.e1-4. 25443857 10.1016/j.jalz.2014.07.003 PMC4300247

[58] Hanseeuw BJ , Malotaux V, Dricot L, et al Defining a Centiloid scale threshold predicting long-term progression to dementia in patients attending the memory clinic: An [(18)F] flutemetamol amyloid PET study. Eur J Nucl Med Mol Imaging. 2021;48:302–310. 32601802 10.1007/s00259-020-04942-4 PMC7835306

[59] Tucholka A , Fritsch V, Poline J-B, Thirion B. An empirical comparison of surface-based and volume-based group studies in neuroimaging. Neuroimage. 2012;63:1443–1453. 22732555 10.1016/j.neuroimage.2012.06.019

[60] Fleisher AS , Pontecorvo MJ, Devous MD Sr, et al Positron emission tomography imaging with [18F]flortaucipir and postmortem assessment of Alzheimer disease neuropathologic changes. JAMA Neurol. 2020;77:829–839. 32338734 10.1001/jamaneurol.2020.0528 PMC7186920

[61] Pontecorvo MJ , Devous MD Sr, Navitsky M, et al Relationships between flortaucipir PET tau binding and amyloid burden, clinical diagnosis, age and cognition. Brain. 2017;140:748–763. 28077397 10.1093/brain/aww334 PMC5382945

[62] Buckner RL , Andrews-Hanna JR, Schacter DL. The brain's default network: Anatomy, function, and relevance to disease. Ann N Y Acad Sci. 2008;1124:1–38. 18400922 10.1196/annals.1440.011

[63] Touroutoglou A , Katsumi Y, Brickhouse M, et al The sporadic early-onset Alzheimer's disease signature of atrophy: Preliminary findings from the Longitudinal Early-onset Alzheimer's Disease Study (LEADS) cohort. Alzheimers Dement. 2023;19 Suppl 9(Suppl 9):S74–S88. 37850549 10.1002/alz.13466 PMC10829523

[64] Raichle ME . The brain's default mode network. Annu Rev Neurosci. 2015;38:433–447. 25938726 10.1146/annurev-neuro-071013-014030

[65] Dickerson BC , Stoub TR, Shah RC, et al Alzheimer-signature MRI biomarker predicts AD dementia in cognitively normal adults. Neurology. 2011;76:1395–1402. 21490323 10.1212/WNL.0b013e3182166e96 PMC3087406

[66] Braak H , Alafuzoff I, Arzberger T, Kretzschmar H, Del Tredici K. Staging of Alzheimer disease-associated neurofibrillary pathology using paraffin sections and immunocytochemistry. Acta Neuropathol. 2006;112:389–404. 16906426 10.1007/s00401-006-0127-z PMC3906709

[67] Zanotti-Fregonara P , Pascual B, Rizzo G, et al Head-to-head comparison of 11C-PBR28 and 18F-GE180 for quantification of the translocator protein in the human brain. J Nucl Med. 2018;59:1260–1266. 29348317 10.2967/jnumed.117.203109

[68] Leng F , Hinz R, Gentleman S, et al Neuroinflammation is independently associated with brain network dysfunction in Alzheimer's disease. Mol Psychiatry. 2023;28:1303–1311. 36474000 10.1038/s41380-022-01878-z PMC10005956

[69] Terada T , Yokokura M, Obi T, et al In vivo direct relation of tau pathology with neuroinflammation in early Alzheimer's disease. J Neurol. 2019;266:2186–2196. 31139959 10.1007/s00415-019-09400-2

[70] Rauchmann BS , Brendel M, Franzmeier N, et al Microglial activation and connectivity in Alzheimer disease and aging. Ann Neurol. 2022;92:768–781. 36053756 10.1002/ana.26465

[71] Finze A , Biechele G, Rauchmann BS, et al Individual regional associations between Aβ-, tau- and neurodegeneration (ATN) with microglial activation in patients with primary and secondary tauopathies. Mol Psychiatry. 2023;28:4438–4450. 37495886 10.1038/s41380-023-02188-8 PMC10827660

[72] Gottfried-Blackmore A , Sierra A, Jellinck PH, McEwen BS, Bulloch K. Brain microglia express steroid-converting enzymes in the mouse. J Steroid Biochem Mol Biol. 2008;109(1–2):96–107. 18329265 10.1016/j.jsbmb.2007.12.013 PMC2423427

[73] Kouri N , Frankenhauser I, Peng Z, et al Clinicopathologic heterogeneity and glial activation patterns in Alzheimer disease. JAMA Neurol. 2024;81:619–629. 38619853 10.1001/jamaneurol.2024.0784 PMC11019448

[74] Whitwell JL , Dickson DW, Murray ME, et al Neuroimaging correlates of pathologically defined subtypes of Alzheimer's disease: A case-control study. Lancet Neurol. 2012;11:868–877. 22951070 10.1016/S1474-4422(12)70200-4 PMC3490201

[75] Pascual B , Funk Q, Zanotti-Fregonara P, et al Neuroinflammation is highest in areas of disease progression in semantic dementia. Brain. 2021;144:1565–1575. 33824991 10.1093/brain/awab057 PMC13016666

[76] Montal V , Vilaplana E, Pegueroles J, et al Biphasic cortical macro- and microstructural changes in autosomal dominant Alzheimer's disease. Alzheimers Dement. 2021;17:618–628. 33196147 10.1002/alz.12224 PMC8043974

[77] Serrano-Pozo A , Mielke ML, Gomez-Isla T, et al Reactive glia not only associates with plaques but also parallels tangles in Alzheimer's disease. Am J Pathol. 2011;179:1373–1384. 21777559 10.1016/j.ajpath.2011.05.047 PMC3157187

[78] Li J , Curley WH, Guerin B, et al Mapping the subcortical connectivity of the human default mode network. Neuroimage. 2021;245:118758. 34838949 10.1016/j.neuroimage.2021.118758 PMC8945548

[79] Grothe MJ , Sepulcre J, Gonzalez-Escamilla G, et al Molecular properties underlying regional vulnerability to Alzheimer's disease pathology. Brain. 2018;141:2755–2771. 30016411 10.1093/brain/awy189 PMC6113636

[80] Ngo GN , Hori Y, Everling S, Menon RS. Joint-embeddings reveal functional differences in default-mode network architecture between marmosets and humans. Neuroimage. 2023;272:120035. 36948281 10.1016/j.neuroimage.2023.120035

[81] Garin CM , Hori Y, Everling S, et al An evolutionary gap in primate default mode network organization. Cell Rep. 2022;39:110669. 35417698 10.1016/j.celrep.2022.110669 PMC9088817

[82] Whittington A , Sharp DJ, Gunn RN; Alzheimer’s Disease Neuroimaging Initiative. Spatiotemporal distribution of β-amyloid in Alzheimer disease is the result of heterogeneous regional carrying capacities. J Nucl Med. 2018;59:822–827. 29146694 10.2967/jnumed.117.194720 PMC5932528

[83] LaPoint MR , Baker SL, Landau SM, Harrison TM, Jagust WJ. Rates of β-amyloid deposition indicate widespread simultaneous accumulation throughout the brain. Neurobiol Aging. 2022;115:1–11. 35447369 10.1016/j.neurobiolaging.2022.03.005 PMC9986974

[84] Jacobs HIL , Hedden T, Schultz AP, et al Structural tract alterations predict downstream tau accumulation in amyloid-positive older individuals. Nat Neurosci. 2018;21:424–431. 29403032 10.1038/s41593-018-0070-z PMC5857215

[85] Vogel JW , Iturria-Medina Y, Strandberg OT, et al Spread of pathological tau proteins through communicating neurons in human Alzheimer's disease. Nat Commun. 2020;11:2612. 32457389 10.1038/s41467-020-15701-2 PMC7251068

[86] Asai H , Ikezu S, Tsunoda S, et al Depletion of microglia and inhibition of exosome synthesis halt tau propagation. Nat Neurosci. 2015;18:1584–1593. 26436904 10.1038/nn.4132 PMC4694577

[87] Ising C , Venegas C, Zhang S, et al NLRP3 inflammasome activation drives tau pathology. Nature. 2019;575:669–673. 31748742 10.1038/s41586-019-1769-z PMC7324015

[88] Ossenkoppele R , Leuzy A, Cho H, et al The impact of demographic, clinical, genetic, and imaging variables on tau PET status. Eur J Nucl Med Mol Imaging. 2021;48:2245–2258. 33215319 10.1007/s00259-020-05099-w PMC8131404

[89] Doré V , Krishnadas N, Bourgeat P, et al Relationship between amyloid and tau levels and its impact on tau spreading. Eur J Nucl Med Mol Imaging. 2021;48:2225–2232. 33495928 10.1007/s00259-021-05191-9 PMC8175299

[90] Böttcher C , Schlickeiser S, Sneeboer MAM, et al Human microglia regional heterogeneity and phenotypes determined by multiplexed single-cell mass cytometry. Nat Neurosci. 2019;22:78–90. 30559476 10.1038/s41593-018-0290-2

[91] Prokop S , Lee VMY, Trojanowski JQ. Neuroimmune interactions in Alzheimer's disease-new frontier with old challenges? Prog Mol Biol Transl Sci. 2019;168:183–201. 31699314 10.1016/bs.pmbts.2019.10.002 PMC6939624

[92] Van Weehaeghe D , Babu S, De Vocht J, et al Moving toward multicenter therapeutic trials in amyotrophic lateral sclerosis: Feasibility of data pooling using different translocator protein pet radioligands. J Nucl Med. 2020;61:1621–1627. 32169920 10.2967/jnumed.119.241059 PMC9364895

[93] Zanotti-Fregonara P , Kreisl WC, Innis RB, Lyoo CH. Automatic extraction of a reference region for the noninvasive quantification of translocator protein in brain using (11)C-PBR28. J Nucl Med. 2019;60:978–984. 30655330 10.2967/jnumed.118.222927 PMC6604696

[94] Lyoo CH , Ikawa M, Liow JS, et al Cerebellum can serve as a pseudo-reference region in Alzheimer disease to detect neuroinflammation measured with PET radioligand binding to translocator protein. J Nucl Med. 2015;56:701–706. 25766898 10.2967/jnumed.114.146027 PMC4839390

[95] Garland EF , Dennett O, Lau LC, et al The mitochondrial protein TSPO in Alzheimer's disease: Relation to the severity of AD pathology and the neuroinflammatory environment. J Neuroinflammation. 2023;20:186. 37580767 10.1186/s12974-023-02869-9 PMC10424356

[96] Yasuno F , Kimura Y, Ogata A, et al Kinetic modeling and non-invasive approach for translocator protein quantification with (11)C-DPA-713. Nucl Med Biol. 2022;108–109:76–84. 10.1016/j.nucmedbio.2022.02.005 35349913

[97] Srinivasan K , Friedman BA, Etxeberria A, et al Alzheimer's patient microglia exhibit enhanced aging and unique transcriptional activation. Cell Rep. 2020;31:107843. 32610143 10.1016/j.celrep.2020.107843 PMC7422733

[98] Golde TE . Harnessing immunoproteostasis to treat neurodegenerative disorders. Neuron. 2019;101:1003–1015. 30897353 10.1016/j.neuron.2019.02.027 PMC6594693

