# Supplementary material for: Brain inflammation co-localizes highly with tau in mild cognitive impairment due to early-onset Alzheimer’s disease
Source: Brain. 2024 Jul 16;148(1):119–32. doi: 10.1093/brain/awae234 (PMC11706285; doi:10.1093/brain/awae234)
Supplement: awae234_Supplementary_Data [file awae234_supplementary_data.pdf]

Brain inflammation co-localizes highly with tau in mild cognitive impairment due to early-onset Alzheimer's disease

Johanna Appleton, BA; Quentin Finn, PhD; Paolo Zanotti-Fregonara, MD; Meixiang Yu, PhD; Alireza Faridar, MD; Mohammad O Nakawah, MD; Carlos Zarate, MD; Maria Carrillo, PhD, Bradford C Dickerson, MD; Gil Rabinovici, MD; Liana G Apostolova, MD, Joseph C Masdeu, MD, PhD, and Belen Pascual, PhD

Supplementary Materials

Supplementary Table 1. Additional Neuropsychological Assessments from the National Alzheimer's Coordinating Center (NACC) Unified Data Set (UDS) v3 Battery.

Supplementary Table 2. MRI and PET scanners, protocols, parameters, and number of participants studied in each scanner.

Supplementary Table 3. Regional inflammation and tau differences between MCI-EOAD and CU. ANCOVA *p*-values uncorrected for multiple comparisons in each Hammers' Atlas region used for the study except the cerebellum, which was the reference region for tau SUVR calculation.

Supplementary Figure 1. [^{11}C]ER176 PET. Average V_T values per MCI-EOAD affinity group.

Supplementary Figure 2. Vertex-wise frequency maps for Aβ and tau.

Supplementary Figure 3. Surface-based analyses for inflammation, Aβ, and tau in MCI-EOAD without correction for partial volume effect.

Supplementary Figure 4. Inflammation and tau in the Tau-PET Braak-stage regions without correction for partial volume effect.

Supplementary Figure 5. Hammers' Atlas regions with the greatest inflammation in patients with MCI-EOAD, reaching statistical significance after family-wise error correction for multiple comparisons, and tau values in the same regions.

Supplementary Figure 6. Inflammation, Aβ and tau in caudate nuclei of MCI-EOAD and CU individuals. *P*-values are uncorrected for multiple comparisons.

Supplementary Figure 7. Intra-subject correlations between inflammation, Aβ, tau and volume in each patient with MCI-EOAD. **A.** Data are corrected for partial volume effect. **B.** Data are not corrected for partial volume effect.

Supplementary Table 1. Additional Neuropsychological Assessments from the National Alzheimer's Coordinating Center (NACC) Unified Data Set (UDS) v3 Battery.

	**MCI-EOAD**† (n=21)	**CU**‡
	Mean *(SD)*	Mean *(SD)*
Dementia Screening		
MoCA	16.1 *(5.6)*	26.3 *(2.8)*
Memory		
Craft Story Immediate – Verbatim (/44)	9.2 *(7.3)*	21.8 *(6.6)*
Craft Story Immediate – Paraphrase (/25)	7.1 *(5.0)*	16.1 *(3.9)*
Craft Story Delayed – Verbatim (/44)	6.6 *(5.8)*	19.0 *(6.6)*
Craft Story Delayed – Paraphrase (/25)	5.3 *(5.8)*	15.0 *(4.2)*
Benson Figure – Copy (/17)	11.6 *(4.6)*	15.5 *(1.5)*
Benson Figure – Delay (/17)	5.2 *(3.5)*	11.4 *(3.0)*
Attention/Executive Functioning		
Digit Span Forward – Correct trials (/14)	6.2 *(2.9)*	8.2 *(2.4)*
Digit Span Forward – Longest (/9)	5.4 *(2.1)*	6.6 *(1.3)*
Digit Span Backward – Correct Trials (/14)	3.9 *(2.5)*	7.1 *(2.2)*
Digit Span Backward – Longest (/8)	3.4 *(1.7)*	5.0 *(1.3)*
Semantic Fluency – Animals (1 min)	11.8 *(5.4)*	21.2 *(5.6)*
Semantic Fluency – Vegetables (1 min)	6.6 *(3.8)*	14.8 *(4.3)*
Phonemic Fluency – F (1 min)	9.6 *(4.8)*	14.7 *(4.6)*
Phonemic Fluency – L (1 min)	8.4 *(4.7)*	13.9 *(4.4)*
Trail Making A (seconds)	80.8 *(50.5)*	31.7 *(14.2)*
Trail Making B (seconds)	202.7 *(100.0)*	83.7 *(46.9)*
Language		
MiNT Naming Test (/32)	25.4 *(6.9)*	29.8 *(2.4)*
Regular Word Reading (/15)	14.8 *(1.1)*	15.0 *(0.1)*
Irregular Word Reading (/15)	13.8 *(2.2)*	14.5 *(0.9)*
Semantic Word-Picture Match (/20)	19.9 *(0.4)*	20.0 *(0.8)*
Semantic Associates Test (/16)	15.1 *(1.3)*	16.0 *(0.3)*
Northwest Anagram Test (/10)	6.8 *(2.1)*	9.2 *(1.4)*
Sentence Repetition Test (/5)	3.3 *(1.8)*	4.5 *(0.7)*
Sentence Reading Test (/5)	4.3 *(1.3)*	4.9 *(0.4)*
Noun-Verb Naming Test – Nouns (/16)	15.2 *(2.0)*	15.9 *(0.3)*
Noun-Verb Naming Test – Verbs (/16)	13.5 *(3.0)*	15.9 *(0.4)*

† Additional neuropsychological tests for 21 of the 25 MCI-EOAD patients enrolled in the study.

‡ Means and standard deviations from the UDS3 and FTLD Module v3.0 neuropsychological batteries in cognitively normal participants as of September 2020, publicly available on the NACC website.

CU: Cognitive Unimpaired; MCI-EOAD: Mild cognitive impairment early-onset Alzheimer's disease; *SD*: Standard Deviation.

Supplementary Table 2. MRI and PET scanners, protocols, parameters, and number of participants studied in each scanner.

**3T MRI Systems** *(for Inflammation participants)*	**Protocol**	**T1-Weighted**			**MCI-EOAD /CU** (n)
		**TR** (s)	**TE** (s)	**Flip Angle** (degrees)	
Philips Ingenia	In-House	8.2	3.7	8°	2/6
Siemens Skyra	ADNI 3	2.3	2.98	9°	1/2
Siemens Vida	ADNI 3	2.3	2.98	9°	22/15
**3T MRI Systems** *(for CU Aβ participants)*	**Protocol**	**T1-Weighted**			**MCI-EOAD /CU** (n)
		**TR** (s)	**TE** (s)	**Flip Angle** (degrees)	
Siemens Vida	ADNI 3	2.3	2.98	9°	0/3
Siemens Prisma	ADNI 3	2.3	2.98	9°	0/16
**3T MRI Systems** *(for CU Tau participants)*	**Protocol**	**T1-Weighted**			**MCI-EOAD /CU** (n)
		**TR** (s)	**TE** (s)	**Flip Angle** (degrees)	
Philips Ingenia	In-House	8.2	3.7	8°	0/12
Siemens Vida	ADNI 3	2.3	2.98	9°	0/11
**PET-CT Systems**	**FWHM** ($x_x y_x z$ mm)	**ER176**	**Aβ**	**Flortaucipir**	**Additional Smoothing** ($x_x y_x z$ mm)
		**MCI-EOAD/CU** (n)			
GE Discovery	5.1x5.1x5.6	3/3	5/0	4/2	0.0x0.0x0.0
Philips Gemini	4.7x4.7x4.7	3/6	0/0	0/13	2.0x2.0x3.2
Siemens Biograph	3.5x3.5x3.5	19/14	17/19	17/8	3.7x3.7x4.5

CU: Cognitive unimpaired; FWHM: Full width at half maximum; MCI-EOAD: Mild cognitive impaired early-onset Alzheimer's disease; n: Number of participants; s: Seconds; TE: Echo Time; TR: Repetition Time.

Supplementary Table 3. Regional inflammation and tau differences between MCI-EOAD and CU. ANCOVA significant *p*-values uncorrected for multiple comparisons in each Hammers' Atlas region used for the study except the cerebellum, which was the reference region for tau SUVR calculation.

Region		***P*-value (MCI-EOAD *vs* CU)**			
		**[^{11}C]ER176**		**[^{18}F]flortaucipir**	
		PVC	nonPVC	PVC	nonPVC
Frontal Lobe					
Superior Frontal Gyrus	Right	0.005	0.030	<0.001	<0.001
	Left	0.006	0.036	<0.001	<0.001
Middle Frontal Gyrus	Right	0.001	0.006	<0.001	<0.001
	Left	0.001	0.005	<0.001	<0.001
Inferior Frontal Gyrus	Right	0.004	0.018	<0.001	<0.001
	Left	0.003	0.010	<0.001	<0.001
Orbitofrontal Cortex	Right	0.009	0.015	<0.001	<0.001
	Left	0.012	0.014	<0.001	<0.001
Precentral Cortex	Right	ns	ns	<0.001	<0.001
	Left	ns	ns	<0.001	<0.001
Insula	Right	0.017	0.049	<0.001	<0.001
	Left	0.014	0.038	<0.001	<0.001
Parietal Lobe					
Superior Parietal Gyrus	Right	<0.001	0.008	<0.001	<0.001
	Left	<0.001	0.007	<0.001	<0.001
Inferior Parietal Gyrus	Right	<0.001	0.002	<0.001	<0.001
	Left	<0.001	0.001	<0.001	<0.001
Postcentral Cortex	Right	0.029	0.048	0.008	0.001
	Left	0.043	ns	0.004	0.001
Cingulum					
Subgenual, Presubgenual and Subcallosal Cingulate	Right	ns	ns	ns	0.002
	Left	ns	ns	ns	<0.001
Anterior Cingulum	Right	ns	ns	<0.001	<0.001
	Left	0.039	ns	<0.001	<0.001
Posterior Cingulum	Right	0.002	0.009	<0.001	<0.001
	Left	<0.001	0.008	<0.001	<0.001
Temporal Lobe					
Temporal Pole	Right	0.006	0.018	<0.001	<0.001
	Left	0.013	0.018	<0.001	<0.001
Superior Temporal Gyrus	Right	0.004	0.026	<0.001	<0.001
	Left	0.006	0.018	<0.001	<0.001
Middle and Inferior Temporal Gyrus	Right	<0.001	0.003	<0.001	<0.001
	Left	<0.001	0.002	<0.001	<0.001
Parahippocampal and Fusiform Gyrus	Right	0.006	0.012	<0.001	<0.001
	Left	0.022	0.013	<0.001	<0.001
Posterior Temporal Lobe	Right	<0.001	0.003	<0.001	<0.001
	Left	<0.001	0.002	<0.001	<0.001
Amygdala	Right	0.001	0.002	<0.001	<0.001
	Left	0.004	0.003	<0.001	<0.001
Hippocampus	Right	0.048	0.028	0.005	<0.001
	Left	0.034	0.021	<0.001	<0.001
Occipital Lobe					
Lateral Occipital	Right	0.003	0.006	<0.001	<0.001
	Left	0.001	0.006	<0.001	<0.001
Cuneus	Right	0.020	0.032	<0.001	<0.001
	Left	0.010	0.034	<0.001	<0.001
Lingual Gyrus	Right	0.030	0.025	<0.001	<0.001
	Left	0.023	0.034	<0.001	<0.001

Ns: No statistically significant differences.

ANCOVA: Analysis of covariance; CU: Cognitive unimpaired; n: Number of participants; MCI-EOAD: Mild cognitive impaired early-onset Alzheimer's disease; PVC: Partial volume correction; SUVR: Standardized uptake value ratio.

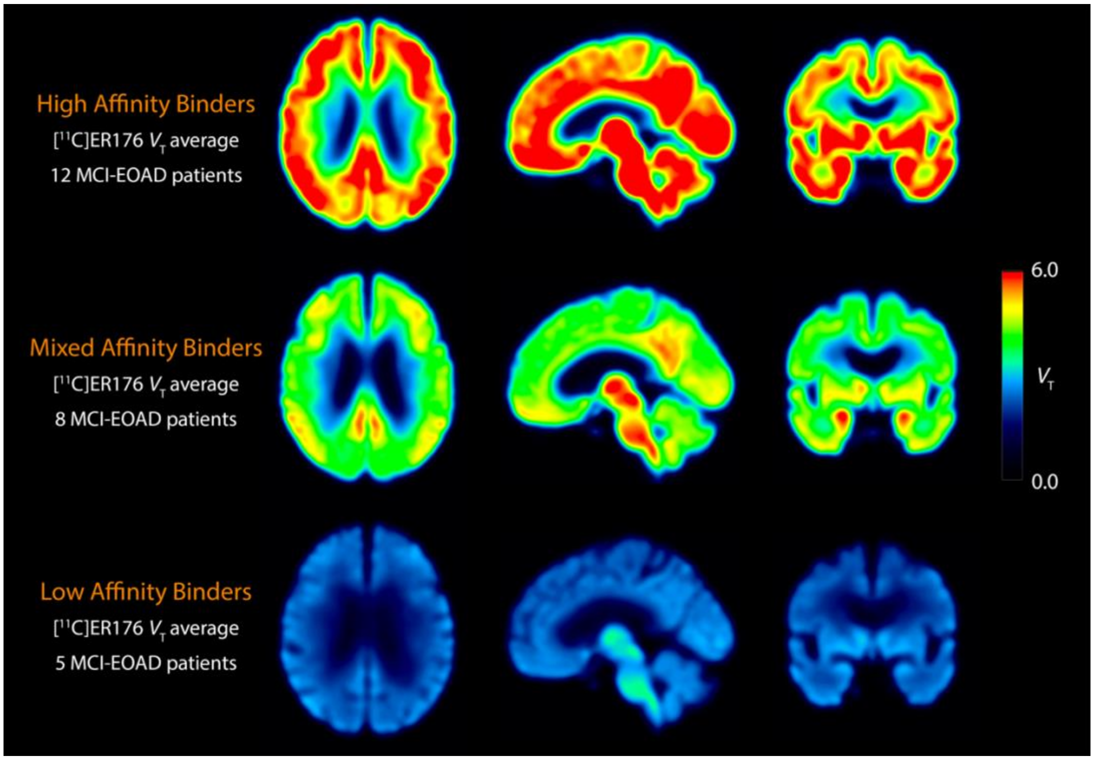

Supplementary Figure 1. [^{11}C]ER176 V_T average per affinity group. MCI-EOAD: Mild cognitive impairment early-onset Alzheimer's disease; V_T: total distribution volume.

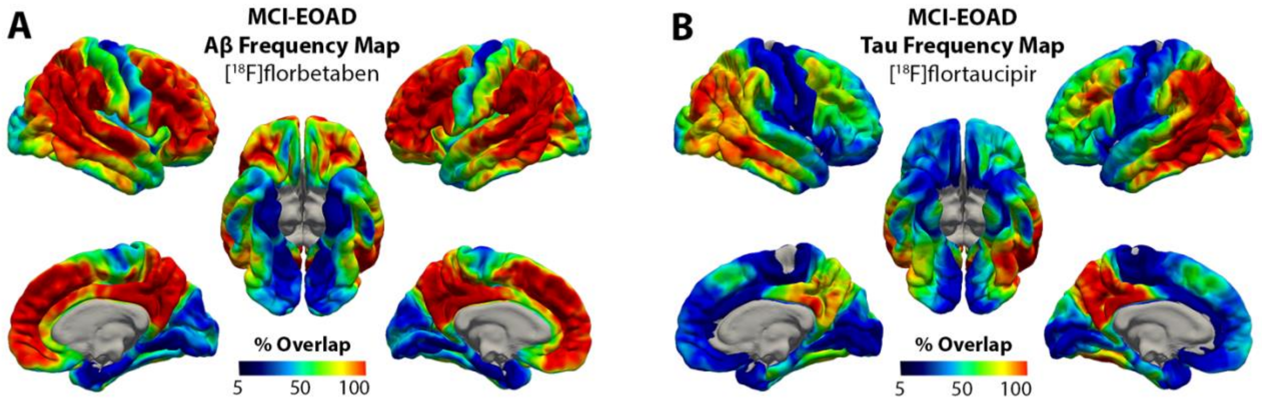

Supplementary Figure 2. Vertex-wise frequency maps for Aβ and tau. **A**: Surface-based frequency of elevated Aβ. Colored vertices indicate the percentage of MCI-EOAD patients (n=21) with elevated [^{18}F]florbetaben uptake. **B**: Surface-based frequency of elevated tau. Colored vertices indicate the percentage of MCI-EOAD patients (n=21) with elevated [^{18}F]flortaucipir uptake. MCI-EOAD: Mild cognitive impairment early-onset Alzheimer's disease.

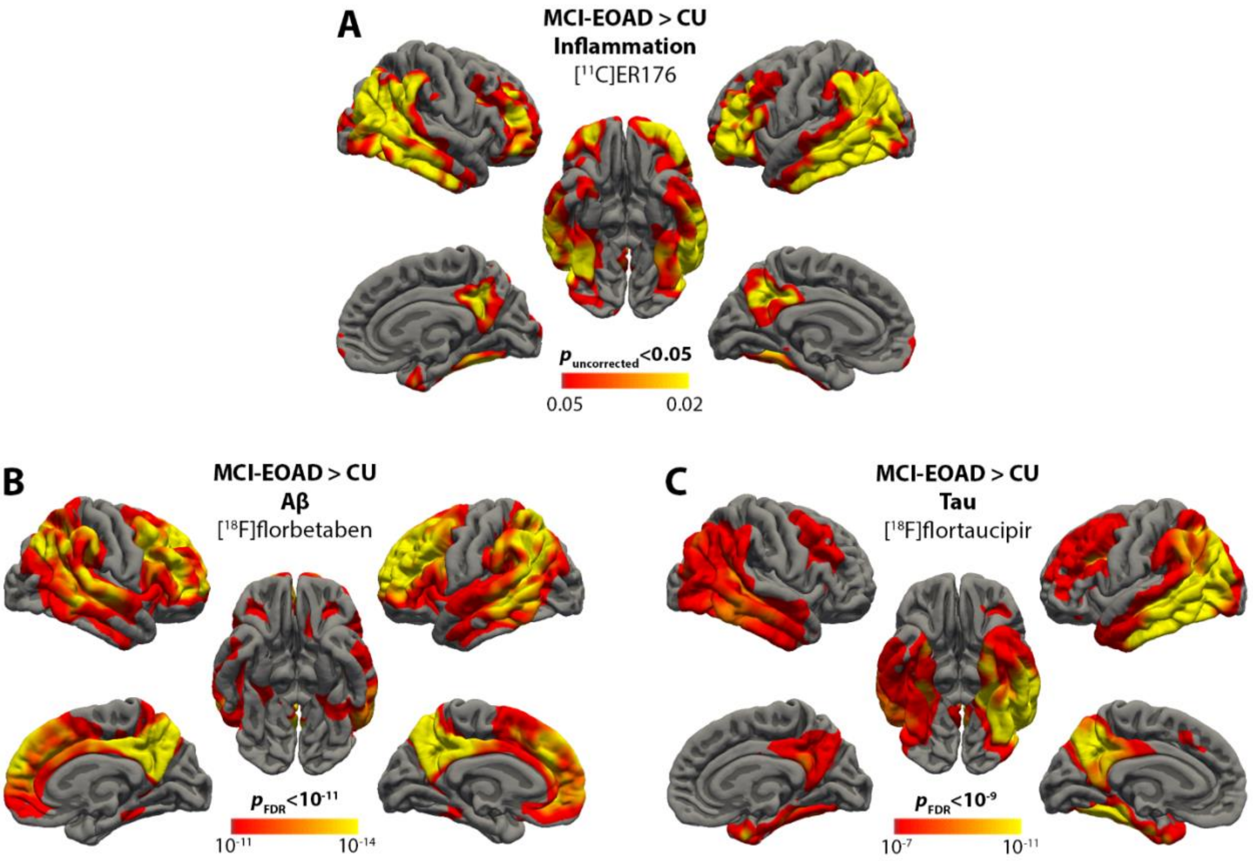

Supplementary Figure 3. Surface-based analyses for inflammation, Aβ, and tau in MCI-EOAD without correction for partial volume effect. CU: Cognitively Unimpaired; FDR: False discovery rate; MCI-EOAD: Mild cognitive impairment early-onset Alzheimer's disease.

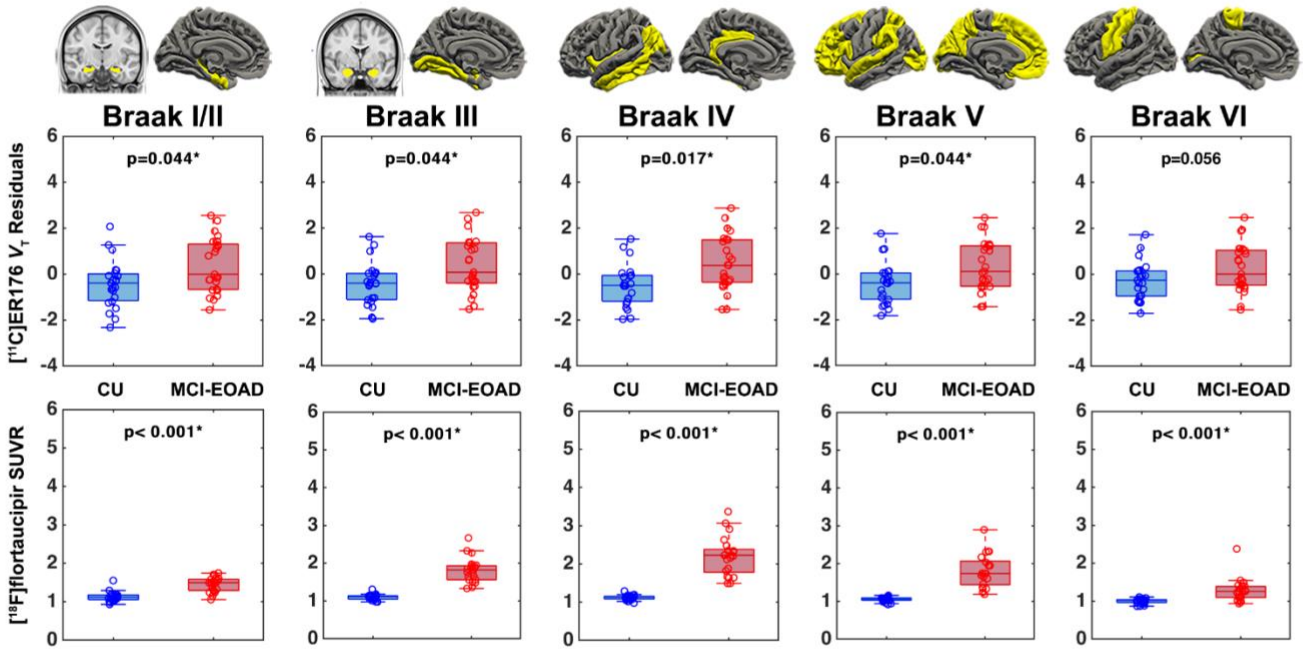

Supplementary Figure 4. Inflammation and tau in the Tau-PET Braak-stage regions without correction for partial volume effect. Box and whisker plots show values of [^{11}C]ER176 (top row) and [^{18}F]flortaucipir (bottom row) uptake in CU controls (in blue) and MCI-EOAD (in red) after FWE correction for multiple comparisons. CU: Cognitive Unimpaired; few: Family-wise error; MCI-EOAD: Mild cognitive impairment early-onset Alzheimer's disease; SUVR: Standardized uptake value ratio; V_T: total distribution volume.

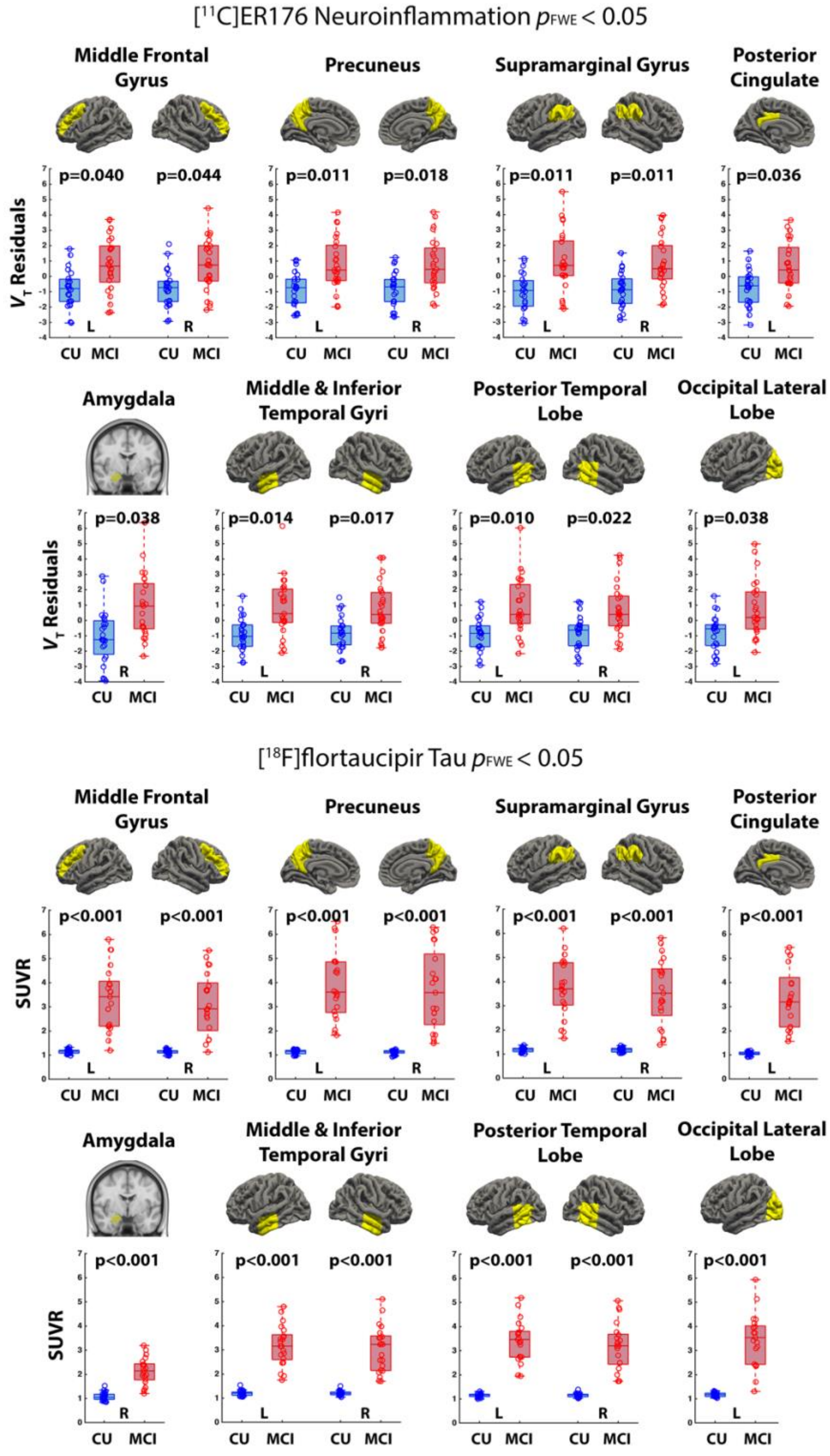

Supplementary Figure 5. Hammers' Atlas regions with the greatest inflammation in patients with MCI-EOAD, reaching statistical significance after family-wise error correction for multiple comparisons (top two rows), and tau values in the same regions (bottom two rows). CU: Cognitive unimpaired; FWE: Family-wise error; MCI: Mild cognitive impaired; SUVR: Standardized uptake value ratio; V_T: Total volume distribution.

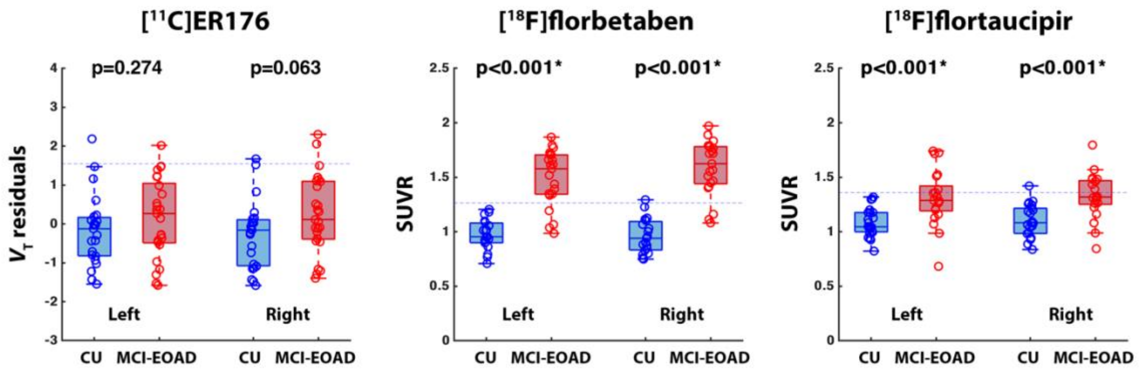

Supplementary Figure 6. Inflammation, Aβ and tau in caudate nuclei of MCI-EOAD and CU individuals. *P*-values are uncorrected for multiple comparisons. CU: Cognitive unimpaired; MCI-EOAD: Mild cognitive impaired early-onset Alzheimer's disease; SUVR: Standardized uptake value ratio; V_T: Total volume distribution.

Supplementary Figure 7A. Intra-subject correlations between inflammation, Aβ, tau and volume in each patient with MCI-EOAD. Data points represent the value of each variable in each of the Hammers' atlas regions. PET data are corrected for partial volume effect.

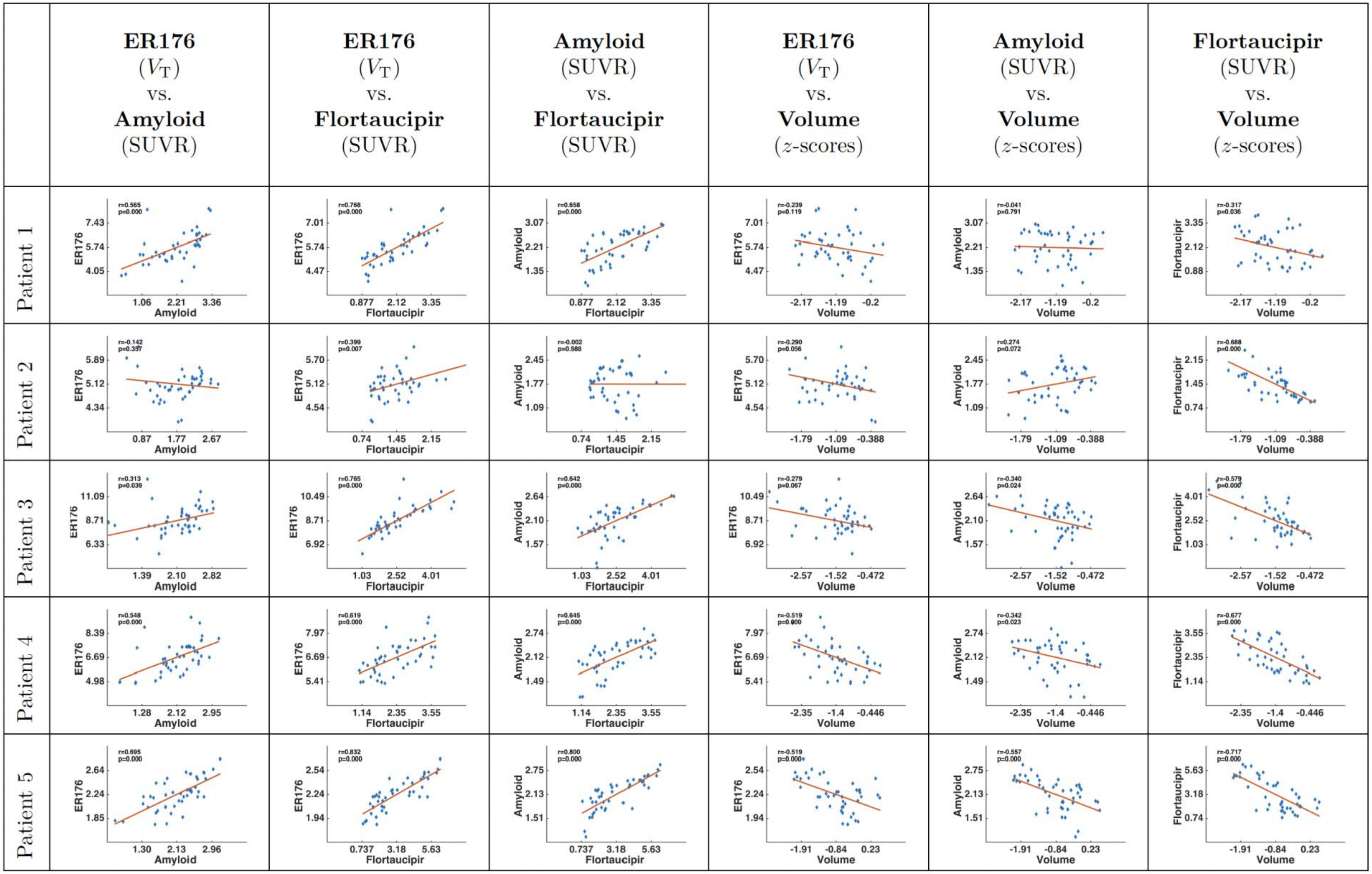

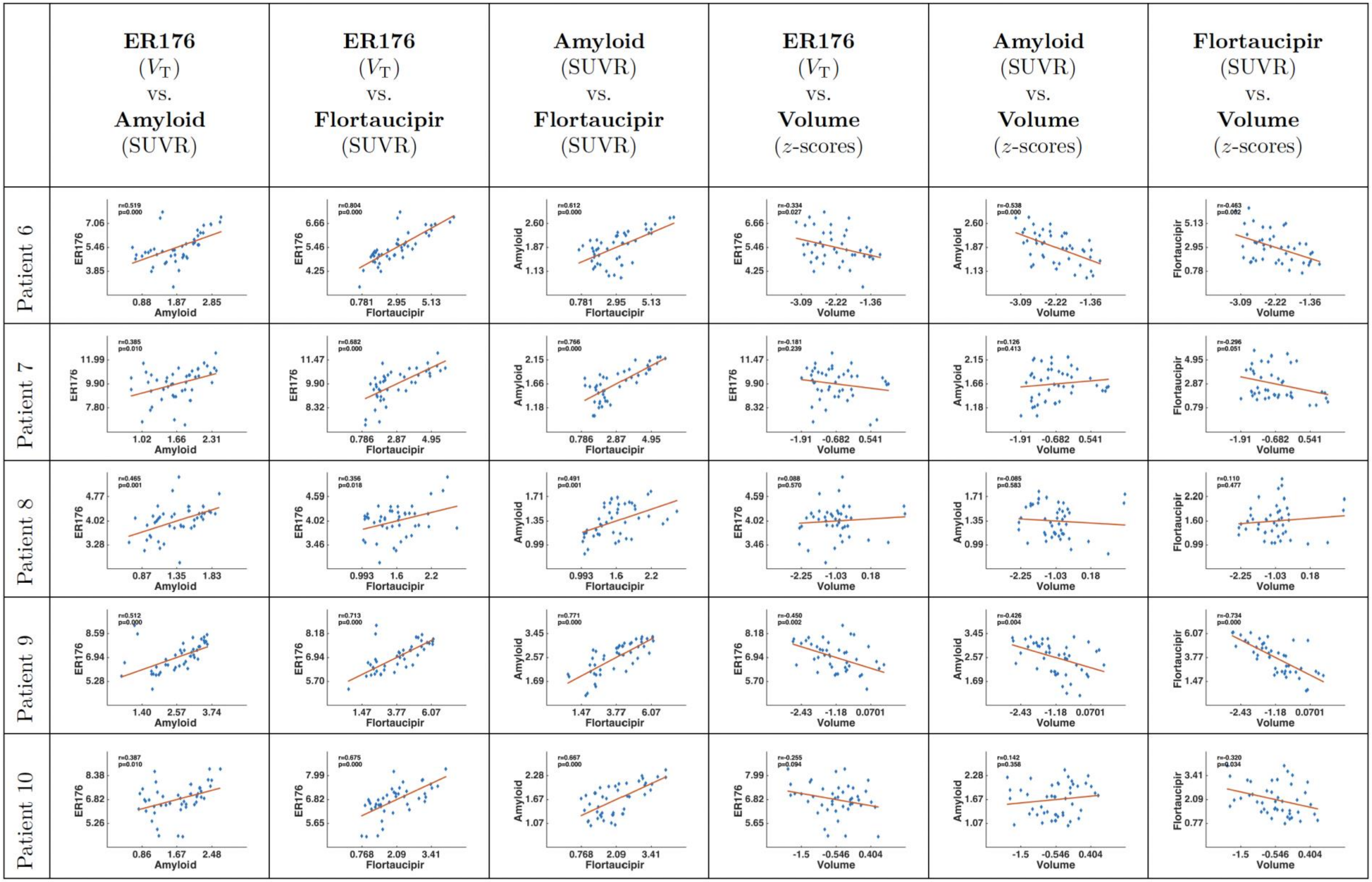
ER176 (V_T) vs. Amyloid (SUVR)
ER176 (V_T) vs. Flortaucipir (SUVR)
Amyloid (SUVR) vs. Flortaucipir (SUVR)
ER176 (V_T) vs. Volume (z-scores)
Amyloid (SUVR) vs. Volume (z-scores)
Flortaucipir (SUVR) vs. Volume (z-scores)
Patient 6
Patient 7
Patient 8
Patient 9
Patient 10

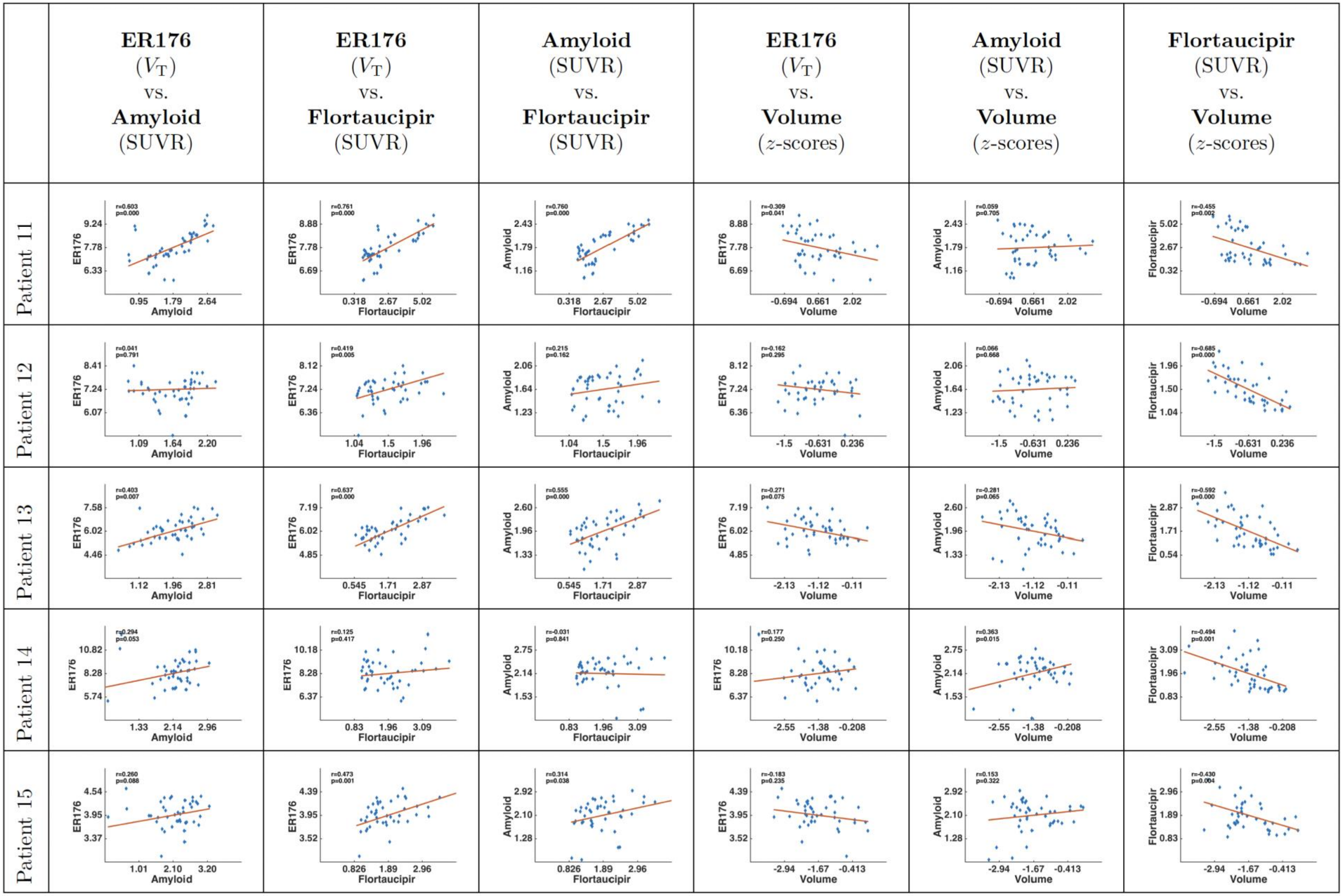
ER176 (V_T) vs. Amyloid (SUVR)
ER176 (V_T) vs. Flortaucipir (SUVR)
Amyloid (SUVR) vs. Flortaucipir (SUVR)
ER176 (V_T) vs. Volume (z-scores)
Amyloid (SUVR) vs. Volume (z-scores)
Flortaucipir (SUVR) vs. Volume (z-scores)
Patient 11
Patient 12
Patient 13
Patient 14
Patient 15

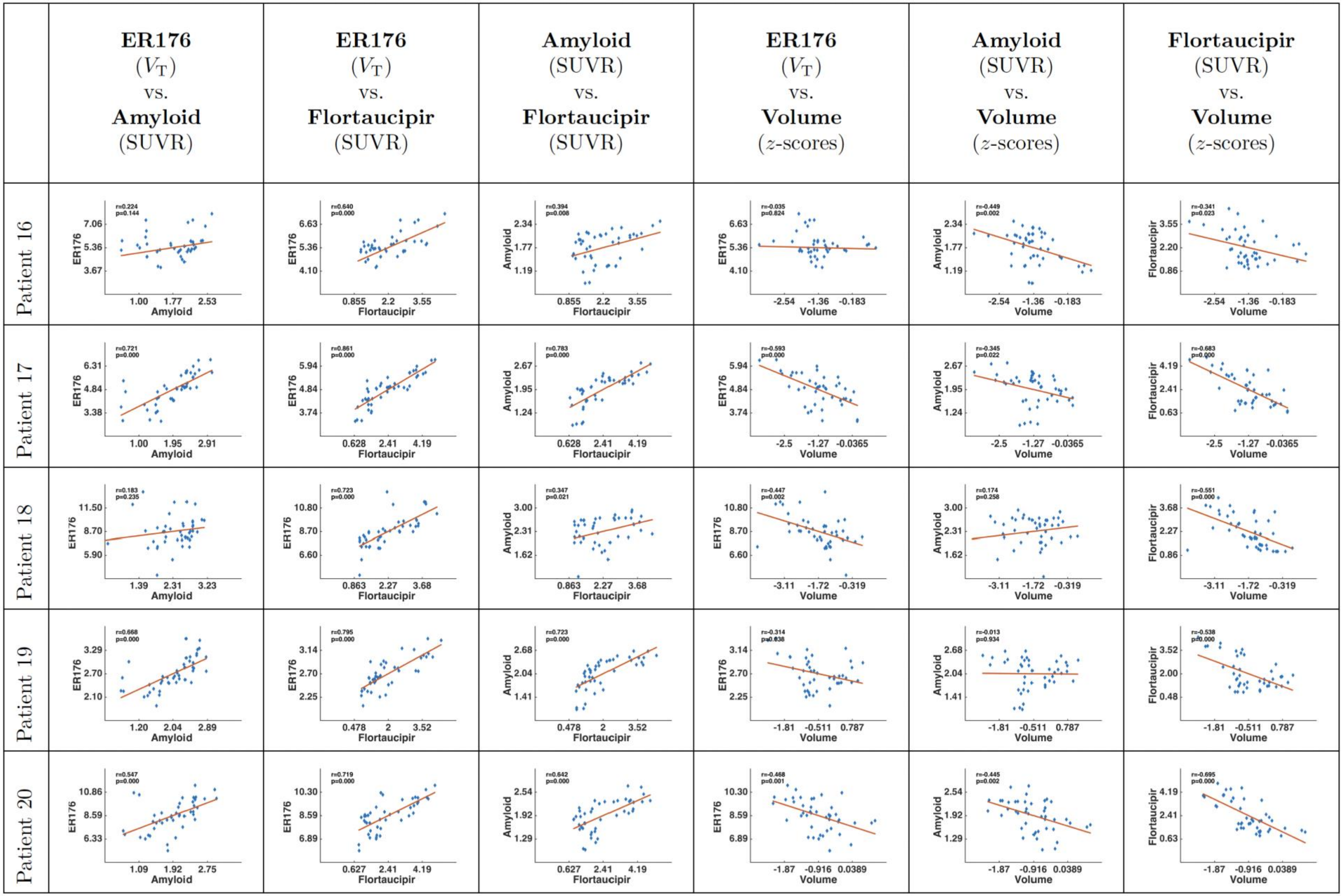
ER176 (VT) vs. Amyloid (SUVR)
ER176 (VT) vs. Flortaucipir (SUVR)
Amyloid (SUVR) vs. Flortaucipir (SUVR)
ER176 (VT) vs. Volume (z-scores)
Amyloid (SUVR) vs. Volume (z-scores)
Flortaucipir (SUVR) vs. Volume (z-scores)
Patient 16
Patient 17
Patient 18
Patient 19
Patient 20

	**ER176** (V_{T}) vs. **Amyloid** (SUVR)	**ER176** (V_{T}) vs. **Flortaucipir** (SUVR)	**Amyloid** (SUVR) vs. **Flortaucipir** (SUVR)	**ER176** (V_{T}) vs. **Volume** (z-scores)	**Amyloid** (SUVR) vs. **Volume** (z-scores)	**Flortaucipir** (SUVR) vs. **Volume** (z-scores)
Patient 21	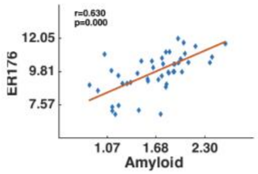	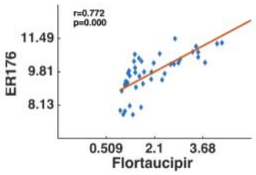	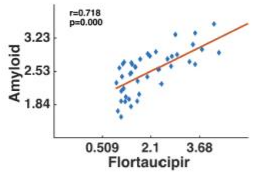	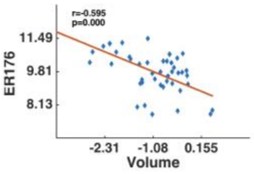	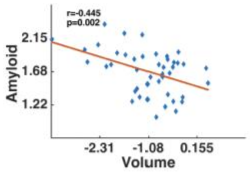	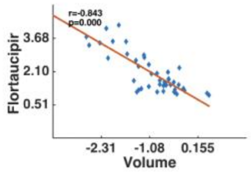
Patient 22	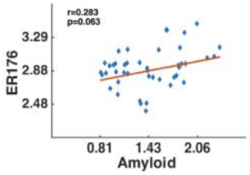	No Flortaucipir Available	No Flortaucipir Available	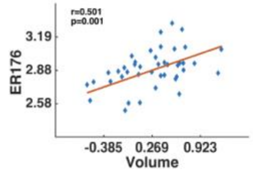	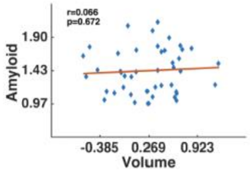	No Flortaucipir Available
Patient 23	No Amyloid Available	No Flortaucipir Available	Neither Tracer Available	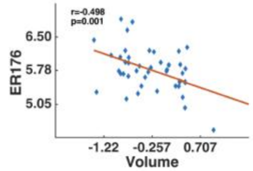	No Amyloid Available	No Flortaucipir Available
Patient 24	No Amyloid Available	No Flortaucipir Available	Neither Tracer Available	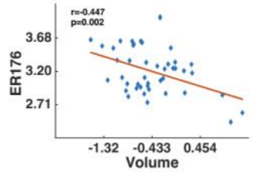	No Amyloid Available	No Flortaucipir Available
Patient 25	No Amyloid Available	No Flortaucipir Available	Neither Tracer Available	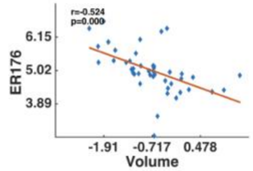	No Amyloid Available	No Flortaucipir Available

Supplementary Figure 7B. Intra-subject correlations between inflammation, Aβ, tau and volume in each patient with MCI-EOAD. Data points represent the value of each variable in each of the Hammers' atlas regions. PET data are not corrected for partial volume effect.

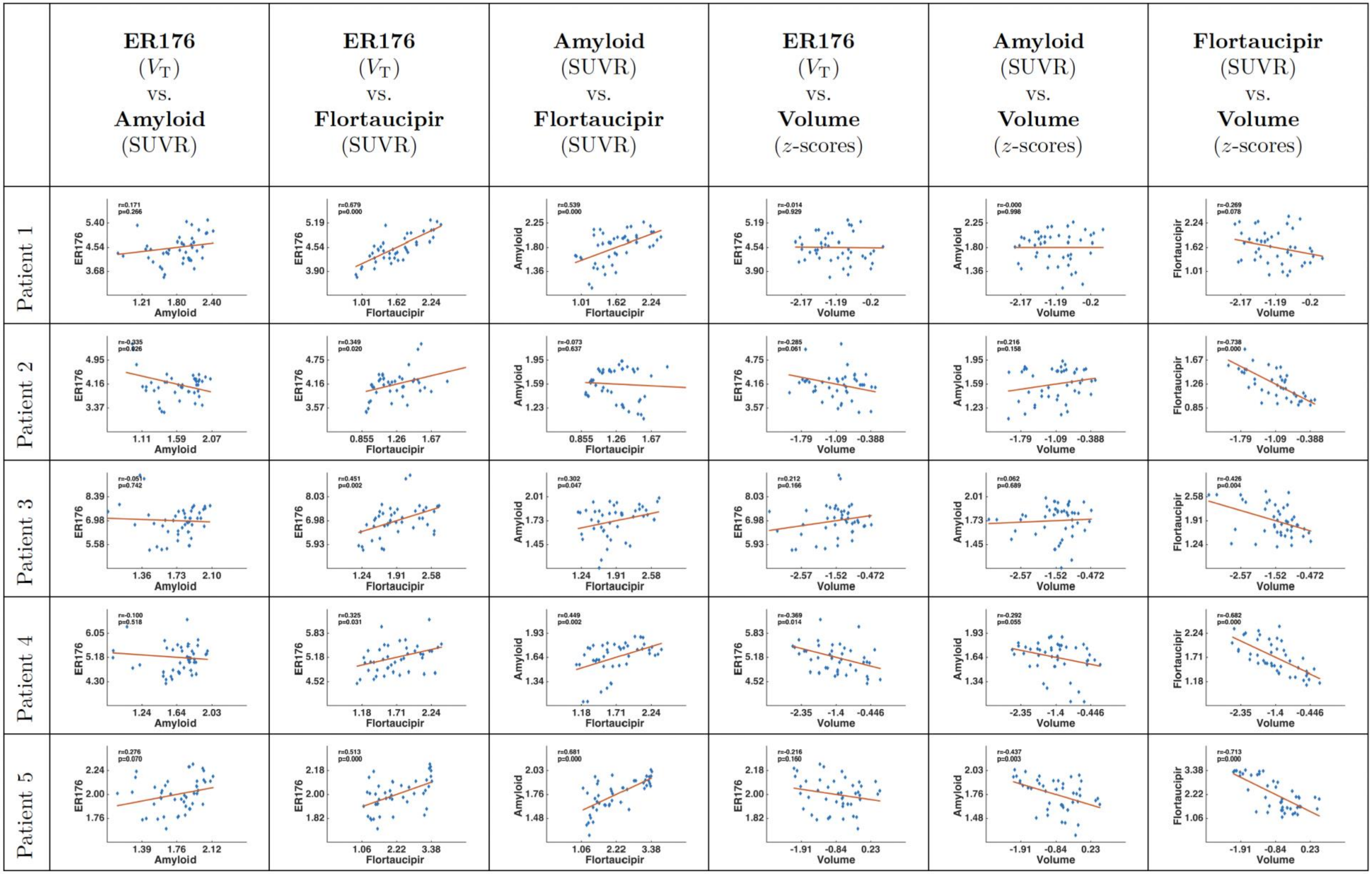

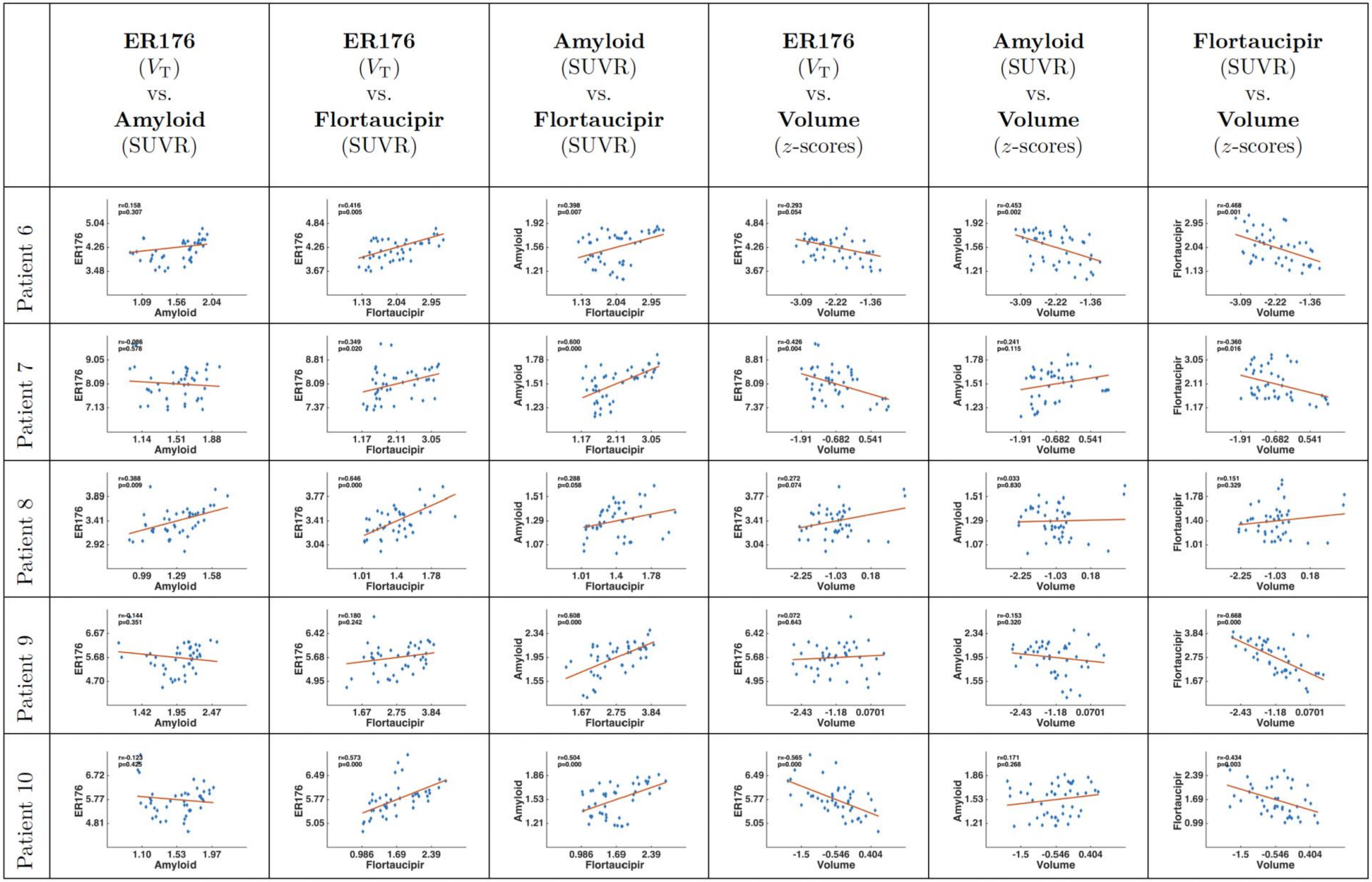
ER176 (V_T) vs. Amyloid (SUVR)
ER176 (V_T) vs. Flortaucipir (SUVR)
Amyloid (SUVR) vs. Flortaucipir (SUVR)
ER176 (V_T) vs. Volume (z-scores)
Amyloid (SUVR) vs. Volume (z-scores)
Flortaucipir (SUVR) vs. Volume (z-scores)
Patient 6
r=0.158 p=0.307
r=0.416 p=0.005
r=0.398 p=0.007
r=-0.293 p=0.054
r=-0.453 p=0.002
r=-0.468 p=0.001
Patient 7
r=-0.086 p=0.578
r=0.349 p=0.020
r=0.600 p=0.000
r=-0.426 p=0.004
r=0.241 p=0.115
r=-0.360 p=0.016
Patient 8
r=0.388 p=0.009
r=0.646 p=0.000
r=0.288 p=0.058
r=0.272 p=0.074
r=0.033 p=0.830
r=0.151 p=0.329
Patient 9
r=-0.144 p=0.351
r=0.180 p=0.242
r=0.608 p=0.000
r=0.072 p=0.643
r=-0.153 p=0.320
r=-0.668 p=0.000
Patient 10
r=-0.123 p=0.425
r=0.573 p=0.000
r=0.504 p=0.000
r=-0.565 p=0.000
r=0.171 p=0.268
r=-0.434 p=0.003
ER176
Amyloid
Flortaucipir
Volume

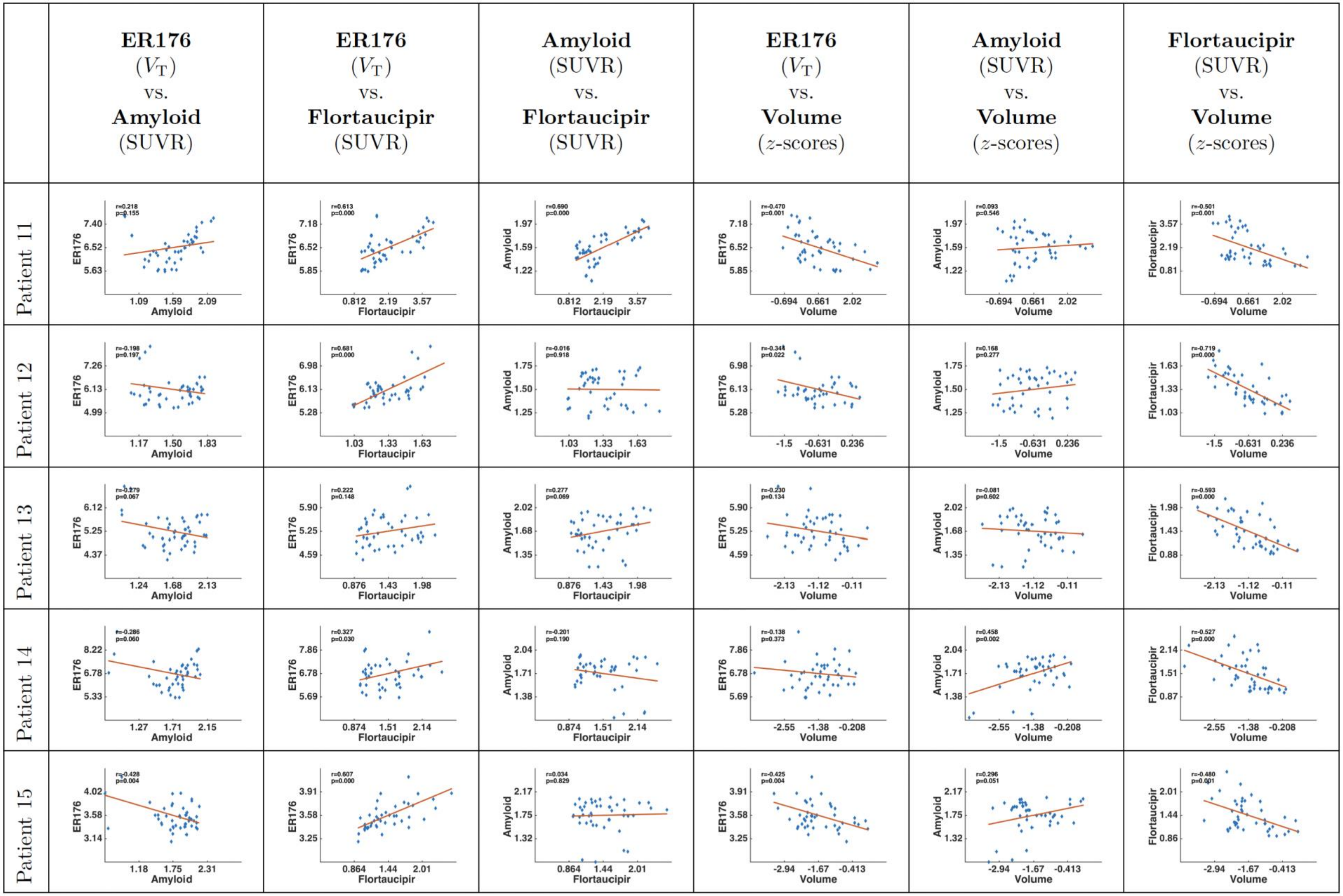
ER176 (V_T) vs. Amyloid (SUVR)
ER176 (V_T) vs. Flortaucipir (SUVR)
Amyloid (SUVR) vs. Flortaucipir (SUVR)
ER176 (V_T) vs. Volume (*z*-scores)
Amyloid (SUVR) vs. Volume (*z*-scores)
Flortaucipir (SUVR) vs. Volume (*z*-scores)
Patient 11
Patient 12
Patient 13
Patient 14
Patient 15

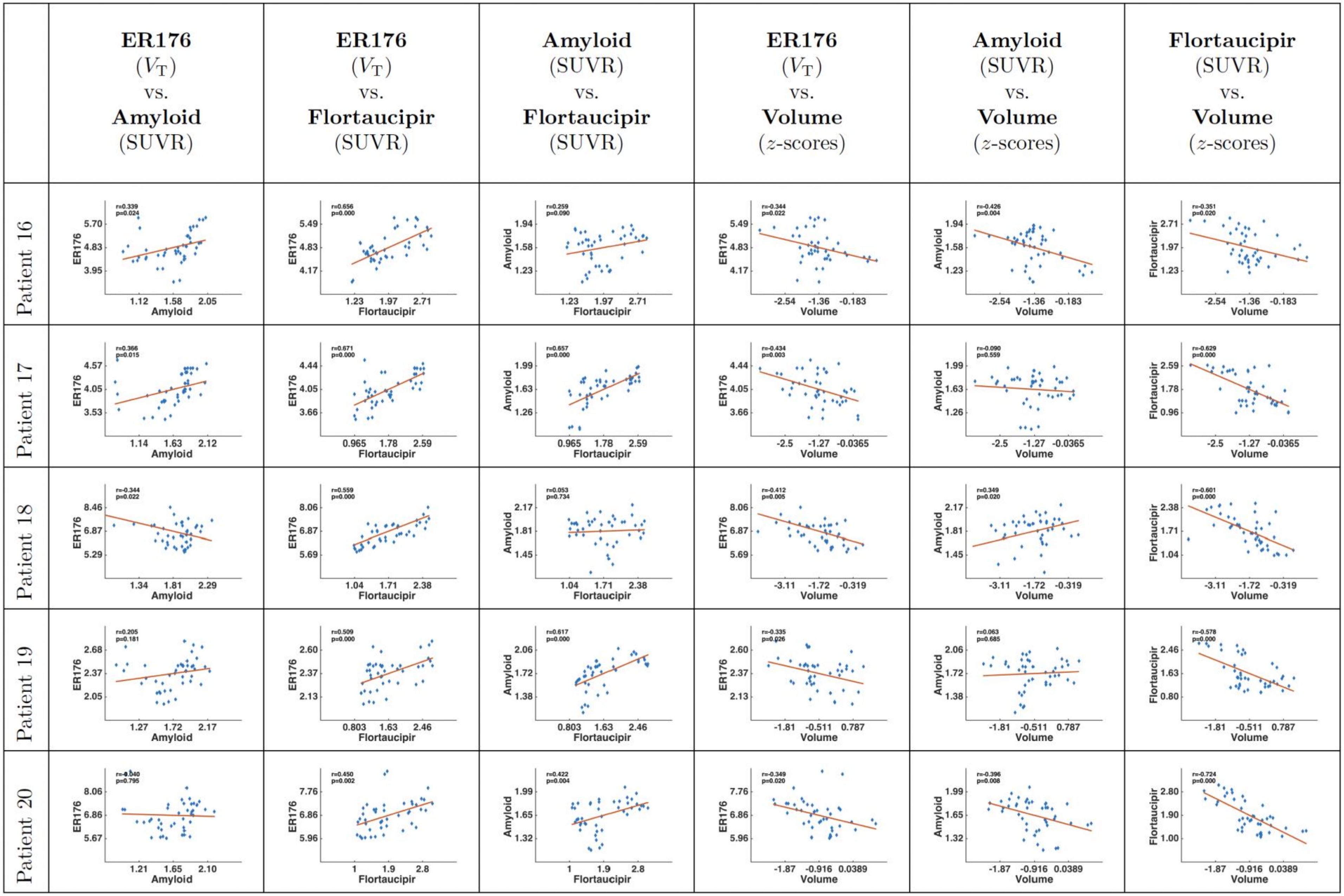
ER176 (V_T) vs. Amyloid (SUVR)
ER176 (V_T) vs. Flortaucipir (SUVR)
Amyloid (SUVR) vs. Flortaucipir (SUVR)
ER176 (V_T) vs. Volume (z-scores)
Amyloid (SUVR) vs. Volume (z-scores)
Flortaucipir (SUVR) vs. Volume (z-scores)
Patient 16
r=0.339 p=0.024
r=0.656 p=0.000
r=0.259 p=0.090
r=-0.344 p=0.022
r=-0.426 p=0.004
r=-0.351 p=0.020
Patient 17
r=0.366 p=0.015
r=0.671 p=0.000
r=0.657 p=0.000
r=-0.434 p=0.003
r=-0.090 p=0.559
r=-0.629 p=0.000
Patient 18
r=-0.344 p=0.022
r=0.559 p=0.000
r=0.053 p=0.734
r=-0.412 p=0.005
r=0.349 p=0.020
r=-0.601 p=0.000
Patient 19
r=0.205 p=0.181
r=0.509 p=0.000
r=0.617 p=0.000
r=-0.335 p=0.026
r=0.063 p=0.685
r=-0.578 p=0.000
Patient 20
r=-0.040 p=0.795
r=0.450 p=0.002
r=0.422 p=0.004
r=-0.349 p=0.020
r=-0.396 p=0.008
r=-0.724 p=0.000
ER176
Amyloid
Flortaucipir
Volume

	**ER176** (V_T) vs. **Amyloid** (SUVR)	**ER176** (V_T) vs. **Flortaucipir** (SUVR)	**Amyloid** (SUVR) vs. **Flortaucipir** (SUVR)	**ER176** (V_T) vs. **Volume** (z-scores)	**Amyloid** (SUVR) vs. **Volume** (z-scores)	**Flortaucipir** (SUVR) vs. **Volume** (z-scores)
Patient 21	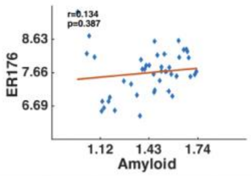	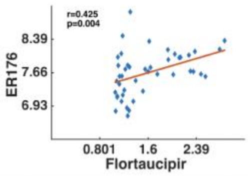	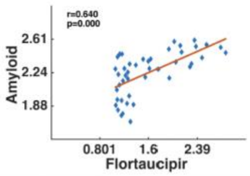	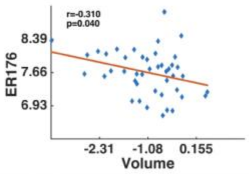	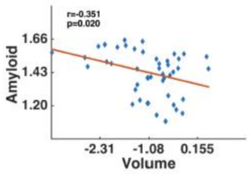	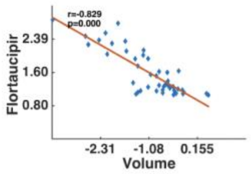
Patient 22	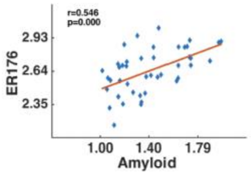	No Flortaucipir Available	No Flortaucipir Available	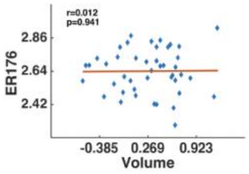	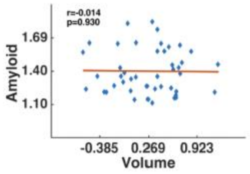	No Flortaucipir Available
Patient 23	No Amyloid Available	No Flortaucipir Available	Neither Tracer Available	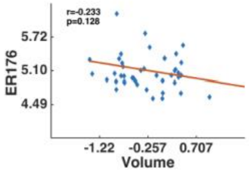	No Amyloid Available	No Flortaucipir Available
Patient 24	No Amyloid Available	No Flortaucipir Available	Neither Tracer Available	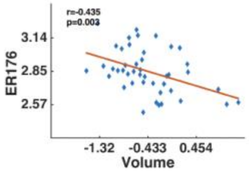	No Amyloid Available	No Flortaucipir Available
Patient 25	No Amyloid Available	No Flortaucipir Available	Neither Tracer Available	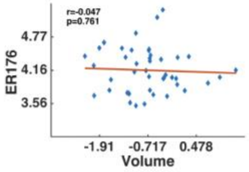	No Amyloid Available	No Flortaucipir Available
